# Environmental and Health Sustainability of the Mediterranean Diet: A Systematic Review

**DOI:** 10.1016/j.advnut.2024.100322

**Published:** 2024-10-18

**Authors:** Victoria Lorca-Camara, Marina Bosque-Prous, Maira Bes-Rastrollo, Cristina O'Callaghan-Gordo, Anna Bach-Faig

**Affiliations:** 1 Faculty of Health Sciences, Universitat Oberta de Catalunya (UOC), Barcelona, Spain; 2 Epi4health Research Group, Faculty of Health Sciences, Universitat Oberta de Catalunya (UOC), Barcelona, Spain; 3 Departament de Psicobiologia i Metodologia en Ciències de la Salut, Universitat Autònoma de Barcelona (UAB), Bellaterra, Spain; 4 CIBERobn, Instituto de Salud Carlos III, Madrid, Spain; 5 IdiSNa, Navarra Institute for Health Research, Pamplona, Spain; 6 Department of Preventive Medicine and Public Health, University of Navarra, Pamplona, Spain; 7 ISGlobal, Barcelona, Spain; 8 Universitat Pompeu Fabra (UPF), Barcelona, Spain; 9 CIBER Epidemiología y Salud Pública (CIBERESP), Madrid, Spain; 10 FoodLab Research Group (2021 SGR 01357), Faculty of Health Sciences, Universitat Oberta de Catalunya (UOC), Barcelona, Spain

**Keywords:** Mediterranean diet, sustainable diet, healthy diet, carbon footprint, water footprint, land use, energy use, dietary environmental impact, food sustainability

## Abstract

The Mediterranean diet (MD) has been shown to be a healthy dietary pattern (DP), and lately it is increasingly being studied as a sustainable DP. The aim of this study is to analyze whether the MD is a sustainable and healthy DP accounting for its carbon footprint, water footprint (WF), land use (LU), and/or energy use, based on the existing scientific literature. We conducted a systematic review following PRISMA guidelines and registered in PROSPERO (CRD42022309916). We included 35 studies: 25 modeling studies, 7 cross-sectional, and 3 longitudinal. Twenty-four studies compared the sustainability of the MD with that of other DPs; 21 assessed the sustainability of the MD compared with dietary consumption data; and 9 evaluated the MD's adherence and its environmental impacts. A total of 29 studies analyzed the carbon footprint, 11 the LU, 20 the WF, and 7 the energy use of the MD. Six articles assessed the health aspect of the diet apart from the environmental impact. The MD showed high nutritional quality, ranging between 122 and 178 points on the health score and between 13.51 and 90.6 points on the nutrient-rich food index. Using the results for environmental footprints in the same measurement units, we were able to quantitatively compare the most frequently assessed diets with MD. When compared with other diets, 91% of the studies referred to the MD as a sustainable DP, and most of the articles in which its adherence was assessed obtained an inverse correlation with the environmental footprints. Environmental footprints of the MD ranged from 1.03 to 5.08 kg CO_2_-eq/person-day for greenhouse gas emissions, 257.2–2735.2 L/person-day for WF, and 4–14.8 m^2^/person-day and 2.85–3.32 m^2^∗year/d for LU. In summary, the available evidence suggests that, in general, the MD is a sustainable and healthy DP, which aligns with planetary health.

**Statement of Significance**

The Mediterranean diet (MD), characterized by health benefits, low environmental impact, sociocultural value, and positive local economic returns, represents a sustainable lifestyle model. We have specifically focused on the 4 environmental footprints of greenhouse gas emission, water footprint, land use, and energy use. Through a comprehensive analysis, we compare the MD with other dietary patterns, highlighting its environmental indicators and health dimension.

## Introduction

Global food production is the main cause of environmental change [1]. The type, composition, and quantity of food produced and consumed affect CO_2_ emissions (that is, carbon footprint) [2], land use (LU) (that is, ecological footprint) [3], and water resource demand [that is, water footprint (WF)] [[4], [5]]. Agriculture and production within the food system contribute to 20%–30% of anthropogenic greenhouse gas emissions (GHGE) [6], of which nearly 80% are associated with livestock [7]. The food system is also the leading cause of deforestation, natural resource depletion, LU change, ecosystem degradation, and biodiversity loss [8]. The cultivation of crops to feed people and livestock accounts for 70% of all human freshwater use and is a major source of water pollution [[3], [9]]. Around 40% of the Earth’s total land area is currently agricultural [[10], [11]]. Animal agriculture requires large amounts of land for both animals and producing their feed [[12], [13, [14]]. Although meat production currently accounts for ∼70% of all agricultural land and consumes 35% of the global grain supply [15], it only generates 18% of the calories consumed by the world’s population. The Mediterranean area is one of the regions under the greatest threat to the environment and human activity from global warming [[5], [16]]. Recent studies have suggested that agricultural land in the southern countries will be diminished by flooding from the rise of the Mediterranean Sea and progressive desiccation [[17, [18]].

In the current context of the global environmental and climate crisis [19], it is of the utmost importance to reduce the environmental impact of diet while ensuring the nutritional requirements of an increasing population are met. Thus, the promotion of healthy and sustainable diets is a key objective for planetary health, a concept defined in 2015 as “the achievement of the highest attainable standard of health, well-being, and equity worldwide through judicious attention to the human systems (political, economic, and social) that shape the future of humanity and the Earth’s natural systems that define the safe environmental limits within which humanity can thrive” [[20], [21]].

Sustainable, healthy diets have been defined by the FAO of the United Nations and the WHO [22] as “Dietary Patterns (DPs) that promote all dimensions of individuals’ health and well-being; have low environmental pressure and impact; are accessible, affordable, safe, and equitable; and are culturally acceptable.” Therefore, the major determinants of a sustainable diet fall into 5 categories: agricultural, health, sociocultural, environmental, and socioeconomic [23]. The environmental impact of food production affects terrestrial and marine environments, and what, and how much we eat directly affects what, and how much is produced [24].

The various components of DPs and their effect on human health and environmental parameters have been studied since the end of the last century. The Mediterranean Diet (MD) is characterized by abundant plant foods (fruit, vegetables, breads, other forms of cereals, potatoes, beans, nuts, and seeds), fresh fruit as the typical daily dessert, olive oil as the principal source of fat, dairy products (principally cheese and yogurt), and fish and poultry consumed in low to moderate amounts, 0−4 eggs consumed weekly, and red meat consumed in low amounts [25]. The characteristics of the MD have been represented using a pyramid that not only summarizes the DP with recommendations for the proportion and frequency of food consumption, but also portrays food-related cultural and social activities and lifestyle aspects, including the regular practice of physical activity, culinary activities, adequate rest, and conviviality [[26], [27]]. The MD has been evaluated as a food consumption pattern with sustainability outcomes, incorporating the following components: *1*) well-established major health and nutrition benefits, prevention of chronic diseases, decrease in public health costs, and overall improvement of well-being, *2*) low environmental impacts and biodiversity conservation, reduction of pressure on natural resources, and climate change mitigation, *3*) local economic returns, sustainable territorial development, reduction in rural poverty, and food waste and food loss reduction, *4*) high social and cultural food value and identity, social interaction, and consumer empowerment [[28], [29], [30], [31]].

The Mediterranean region has high agricultural biodiversity with great diversity in food plant species, subspecies, varieties, and cultivars in the MD [[28], [29], [30], [32], [33], [34], [35], [36]]. The Mediterranean basin is characterized by high biodiversity attributed to its location at the intersection of Africa and Eurasia, its topographical diversity, and several social, political, ecological, and climatic factors [[37], [38]]. Furthermore, the MD, as a plant-based DP, presents high agrobiodiversity resulting from the adaptation of plants brought from different geographical locations that became indigenous and further diverged into cultivars [39]. Approximately one-third of the foodstuffs used for feeding the population originated from the Mediterranean basin [40] with a significant contribution to biodiversity protection [[30], [33], [36], [39], [41], [42]].

Although the health benefits of the MD, including the dimension of shared meals or commensality in its definition [27], and its status as a cultural heritage that contributes to local economies [43] have long been acknowledged, various studies have shown it to also be environmentally sustainable [[44], [45], [46], [47], [48]]. However, as this remains a topic of debate, the sustainability of MD merits a systematic review.

Therefore, the main aim of this systematic review is to assess the sustainability of MD by analyzing recent scientific literature on its GHGE, WF, LU, and energy consumption, while also comparing the environmental footprints of MD to other DPs. Additionally, we analyze the health dimension of MD from the articles included in the systematic review.

## Methods

We conducted a systematic review following the PRISMA checklist and registered the protocol in PROSPERO under the code CRD42022309916 [49]. On 30 September 2022, a literature search was conducted on the electronic databases PubMed, Medline ProQuest, and Web of Science. The following search strategy was used: (Sustainab∗ OR Ecologic∗ OR Climate OR Environment∗ OR Weather) AND (Diet∗ OR Food∗ OR Feeding OR “Eating pattern” OR Nutrition OR Intake)) AND (Mediterranean)) AND (“Carbon footprint” OR “Water footprint” OR “Water use” OR “Land use” OR “Energy footprint” OR “Energy use” OR “Greenhouse gas∗” OR “Global warming potential”). All members of the research team agreed on this search strategy. A second review was conducted to include articles that assessed both the health aspects of the MD and some of its environmental impacts simultaneously. This search was performed in the same databases as the first one. The search strategy remained consistent with the first review, with the addition of the keyword “health∗.”

Inclusion criteria encompassed original articles assessing the MD through modeling or either “a priori” or “a posteriori” approaches in cross-sectional or longitudinal studies. These studies were required to offer empirical values or extrapolate them using linear regression for a minimum of one among the following environmental indicators: GHGE, WF, LU, or energy use. Other systematic reviews and literature reviews were excluded. All population types and articles in English or Spanish were included.

Search results were uploaded to Rayyan [50] to select and extract data and remove duplicates. Two researchers conducted the screening of the articles independently. Articles were initially discarded based on the title and/or abstract, to retain only those of potential interest. The full texts of the remaining studies were then accessed and screened for eligibility. The reference lists of selected articles and noneligible systematic reviews that covered the search topic were then scrutinized for additional eligible articles. A third researcher resolved any disagreements between the reviewers by rigorously complying with the PRISMA procedure for data extraction. All references were managed by the reference manager software Mendeley version 2.79.0.

The selected articles were analyzed, and relevant information was extracted and recorded in a spreadsheet. The extracted data included author details, publication year, the geographical context where the MD was defined, study design, MD definition, methodology for analyzing environmental indicators [such as the European Environmentally Extended Input Output Table, Life Cycle Assessment (LCA), Hybrid Input-Output Analysis, WF Assessment, and WF Network], kcal/sex/age when specified, variables or outcomes related to environmental indicators, and the key findings of the study. Supplemental Table 1 presents the food components in accordance with the MD definition.

For each article, we recorded information on the following outcomes related to sustainability: GHGE, WF, LU, and energy use. Carbon footprint is defined as the total amount of GHGE associated with a product throughout its supply chain and is usually expressed in kg of carbon dioxide equivalent (CO_2_-eq) per unit of output [12]. As an indicator of freshwater use from rainfall, surface, and/or groundwater expressed in liters, WF is a measure of direct and indirect water use by a producer and consumer and water resource appropriation [51]. Measured in m^2^, LU is the amount of agricultural land required to produce crops for direct human consumption, for feed, for use in industry and the energy sector, and for the production of packaging material [52]. Energy use is the industrial energy consumption used in the production and/or cooking of a product or process and is measured in MJ/kg of food consumed [[52], [53]]. In those articles, where it was possible, we converted the numerical value of the environmental footprint to the same functional unit to obtain a range of results. We converted kilocalories to 2000 kcal, and compared the GHGE, WF, and LU results for the most common diets in the articles with those of MD.

We evaluated the quality of reporting using the STROBE statement for case-control, cross-sectional, and cohort studies [54] (see Supplemental Tables 2–10). Modeling studies were assessed with a modified critical appraisal checklist developed by USDA’s Center for Nutrition Policy and Promotion for the 2015 Dietary Guidelines Advisory Committee [55]. This tool is used to assess bias in studies of sustainable diets and DPs and, in modeling studies, to extrapolate the progression of clinical outcomes, transform final outcomes from intermediate measures, examine relations between inputs and outputs to apportion resource use, and extrapolate findings from one clinical setting or population to another. To attain a high score, studies must report the following: variables that have been modeled rather than directly observed; what additional variables have been included or excluded; which statistical relations have been assumed; and the evidence that supports these assumptions. Finally, our systematic review was evaluated with another critical appraisal checklist for systematic reviews [56]. Assessments were independently conducted by 2 reviewers, and any discrepancies resolved through discussion. The results of all quality assessments are available in our Supplemental Material.

## Results

### Study characteristics

**FIGURE 1:**
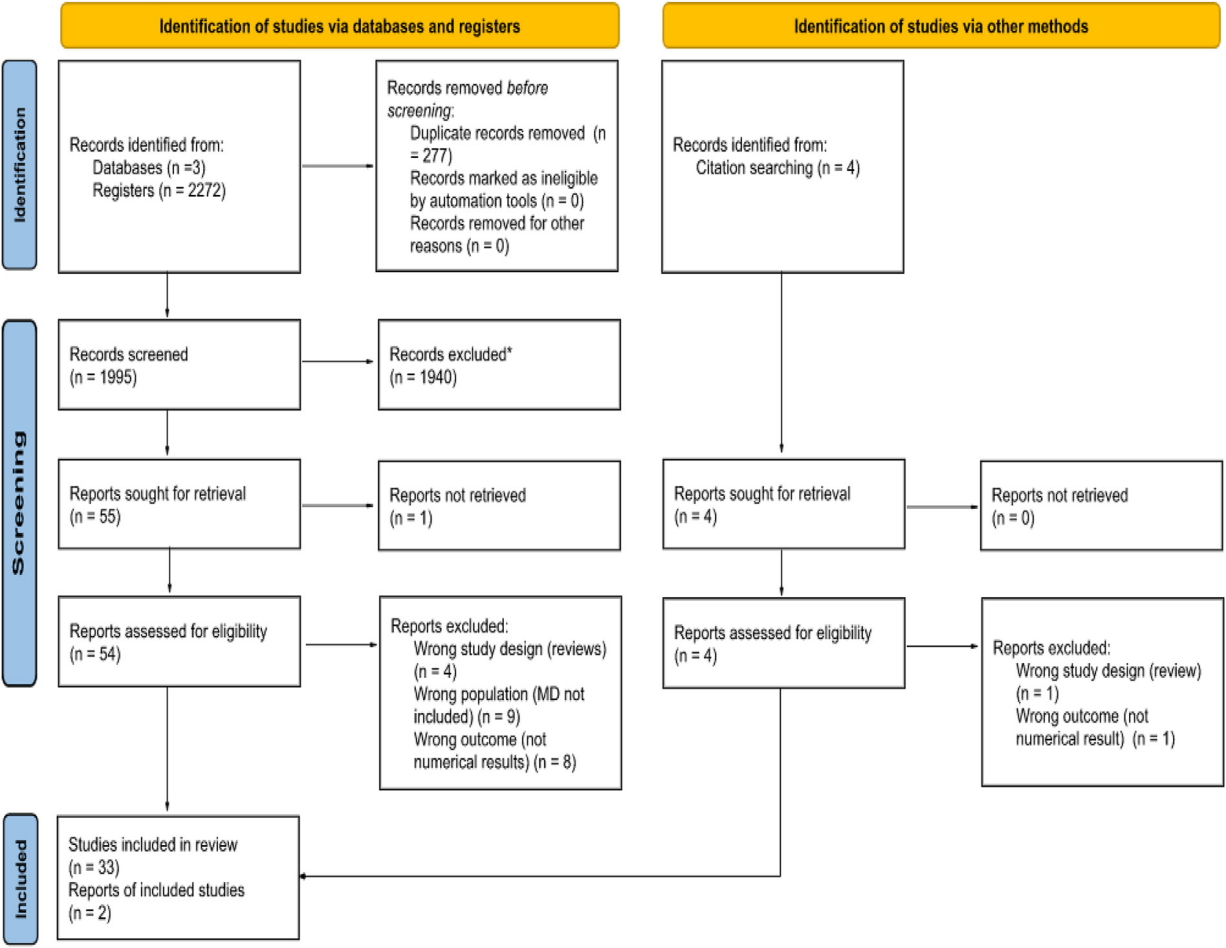
Flowchart of the article selection process. ∗Records excluded based on the title and/or abstract being of no potential interest for this review.

A total of 2272 articles were initially selected from the 3 databases. After duplicates were removed, the titles and abstracts of the remaining 1995 were screened, and 1940 excluded. Most studies eliminated at this stage did not relate to diet or any of the environmental indicators. As 1 article was not available, 54 were accessed for full-text examination. A further 21 studies were excluded due to being revisions of previous studies (*n* = 4), not directly measuring MD (*n* = 9), and providing non-numerical results (*n* = 8). A survey of the references of the remaining 33 articles and the systematic reviews excluded from this study identified 2 additional articles. Overall, 35 articles were selected for systematic review. A flowchart of this selection process is presented in Figure 1. The findings of the selected studies are summarized in Table 1.

**TABLE 1:** Articles included in this systematic review.

First author, year, (ref), study design, and location	MD definition	Method of analysis	kcal/d; sex; age	Variables/outcomes (MD)				Results
				GHGE	Land use	Water footprint	Energy use	
Tukker, 2011, [57], Modeling, European Union	NA	E3IOT model (European Environmentally Extended Input Output Table)	3483.00, adults	1.01E+04 kg CO_2_-eq/y per capita				Dietary recommendations with low red meat, and MD with reduced red meat decreased environmental impacts related to food consumption from 27% to 25% out of all impacts related to final consumption in 27 European Union countries
Sáez-Almendros, 2013, [47], Modeling and data analysis, Spain	Bach-Faig et al., 2011 [26]	Several LCA sources	Around 2000.00, adults	35,510.00 Gg CO_2_-eq/y	8365.00 10^3^ Ha/y	13.20 km^3^/y	239,042.00 TJ/y	Increasing adherence to MD in Spain will reduce GHGE (72%), LU (58%), energy consumption (52%), and WF (33%). Adherence to WD implies an increase in all these descriptors of between 12% and 72%
Wilson, 2013, [58], Modeling and data analysis, New Zealand	Trichopoulou et al., 2009 [59]	Data review: UK data applied to New Zealand, albeit with some approximations	2817.40, M: adults	4.68 kg CO_2_-eq/person-day				Eight scenarios with different characteristics were developed, 2 Asian scenarios and 4 New Zealand scenarios. MD lowered GHGE by 54% when fish were eliminated and emission reduction was optimized, but increased economic cost
Capone, 2013, [60], Modeling and data analysis, Italy	Italian Institute of Food Science of the La Sapienza University-Rome, [61]	Reports of Mekonnen and Hoekstra, 2010 [4]	3649.00, adults			964.29 m^3^/person-year		The WF of the Italian diet (2005–2006) is 69.9% higher than that of MD
Germani, 2014 [62], Modeling and data analysis, Italy	Del Balzo et al., 2012 [63]. Portion size: the new LARN, 2012, [64]. Frequency: Italian pyramid, [61]	Ewing et al., 2010 [65]. JRC-European Platform LCA, 2007 [66]. Hoekstra et al., 2011 [51]	2000.00, not specified	17.04 kg CO_2_-eq/person-week	129.00 m^2^/kg	13,781.00 l/person-week		MD produced 29%, 17.7% and 24.1% lower, respectively, GHGE, WF, and LU than current food consumption of the Italian population (2005–2006)
Van Dooren, 2014 [67], Modeling and data analysis, the Netherlands	Dutch Dietary Guidelines: Health Council, 2006 [68]	LCA: Bellows et al., 2010 [69]. The Agri-footprint method; methodological LCA framework, assumptions and applied data, Blonk et al., 2011 [70]	2000.00, W: 22–50 y	3.41 kg CO_2_-eq/person-day	2.85 m^2^∗ y/d			MD had lower GHGE than average Dutch and official “recommended” Dutch diets, the same as semivegetarian, and more than VEG and VD. MD had higher WF than the last 3 diets and lower than the first 2. MD was generally the health focus option with a higher sustainability score than the current average Dutch, official “recommended” Dutch, semivegetarian, VEG, and VD
Vanham, 2014 [71], Modeling and data analysis, Italy	Bach-Faig et al., 2011 [26]	Approach of Hoekstra et al., 2011 [51]	Not specified			4339.00 l/person-day		Consumption reductions from the WF of Milan (1996–2005) were 29% for MD and 41% for VEG
Tilman, 2014 [72], Modeling, United States	Bach-Faig et al., 2011 [26]. Trichopoulou A et al., 2003 [73]	LCA publications	Not specified	310.00 kg CO_2_-Croplants-eq/person-year				MD, pescetarian and VEG could reduce GHGE from food production to 30%, 45% and 55%, respectively, below those of the projected 2050 income-dependent diet
Pairotti, 2014 [74], Modeling and data analysis, Italy	Modern Diet Mediterranean Food Pyramid, INRAN, [75]	Hybrid input-output analysis – LCA method	Not specified	1.87 tCO_2_-eq/person-year			3814.41 MJ/mo	Reduction of 6.81% GHGE per family on MD vs. the national average diet. VEG had 6.74% lower GHGE than MD. Healthy diet had 4.53% higher GHGE than MD. MD consumes 2.44% less energy than the national average and 4.36% less than the healthy diet. VEG consumes 0.71% less than MD
Vidal, 2015 [76], Modeling, Spain	Diet published by Benidorm Clinical Hospital	LCA database and literature	2000.00, not specified	5.08 kg CO_2_-eq/d				MD was associated with lower environmental impact than diets with more meat, particularly red meat
Van Dooren, 2016 [77], Modeling and data analysis, the Netherlands	Van Dooren et al., 2014 [67]	LCA data: BSI 2008 [78]. JRC 2010 [79]	2503.00, M: 31–50 y	3.24 kg CO_2_-eq/person-day	4.15 m^2^∗ y/d			MD had lower GHGE than new Nordic and presents Dutch diets, the same as historical low lands and higher than dietary guideline and optimized low lands. MD had the same LU as present Dutch diet and was higher than the others
Benvenuti, 2016 [80], Modeling, Italy	Guide of health and nutritional practices: City of Rome, 2013 [81]	Database of the World-Wide Fund for Nature, 2009 [82]	500.00–700.00, children from 5 to 10 y	7.77 kg CO_2_-eq/mo; 10.85 kg CO_2_-eq/mo		16.64 m^3^/mo; 13.72 m^3^/mo		When compared with standard MD school menus defined by nutritionists, a MD menu schedule designed to minimize GHGE would save over 40% in GHGE and over 20% in WF, and a MD menu schedule designed to minimize water usage would save over 35% in WF and over 20% in GHGE
Blas, 2016 [83], Modeling, Spain	Bach-Faig et al., 2011 [26]	Mekonnen and Hoekstra, 2011 [51], 2012 [84]	Not specified			5276.00 L/person-day		The American diet has a 29% higher WF than MD, regardless of product origin
Vanham, 2016 [85], Modeling and data analysis, European cities	Bach-Faig et al., 2011 [26], with city-specific population statics and average daily energy and protein requirements	Mekonnen and Hoekstra, 2011 [51], 2012 [84]	Not specified			3654.00^1^; 2363.00^2^; 3170.00^3^; 3285.00^4^; 2882.00^5^; 2796.00^6^; 2875.00^7^; 2872.00^8^; 2459.00^9^; 3579.00^10^; 3580.00^11^; 3090.00^12^; 3090.00^13^ l/person-day		Compared with actual diets in Mediterranean countries, MD including meat leads to WF reductions of 19%−43%, Pesco-VEG leads to WF reductions of 28%−52%, and VEG leads to WF reductions of 30%−53%
Castañé, 2017, [86], Modeling, Mediterranean Area	Bach-Faig et al., 2011 [26], with weekly diet composed of 7 daily menus	Extensive literature research, including studies using an LCA framework	2025.70, W: moderately active adult	20.00 kg CO_2_-eq/person-week				GHGE were 35% higher for MD than VD, which resulted in 12% higher nutritional quality than MD, although some likely micronutrient deficiencies such as B12 in VD were not assessed
Ulaszewska, 2017 [87], Modeling, Italy	INRAN guidelines, 2003 [75]. Consumption of tap and bottled water: EFSA Comprehensive European Food Consumption Database	LCA studies	2100.00, not specified	23.56 kg CO_2_-eq/wk				GHGE of MD were 8.7% lower than the new Nordic diet, although food distribution and the contribution of individual food categories varied considerably
Fresán, 2018 [88], Longitudinal observational study, Spain	Adh MD (priori): MDS: Trichopoulou et al., 2003 [73]	Sources of secondary data (LCA)	2526.00, specified with the mean	−0.73 kg CO_2_-eq/d	−0.71 m^2^/d	−58.88 L/d	−0.86 MJ/d	Increased adherence to MD was associated with lower LU, WF and GHGE
Murakami, 2018 [89], Cross-sectional study, Japan	Adh MD (priori): –mMDS: Struijk et al., 2014 [90]. Trichopoulou et al., 2005 [91]	LCA data from the literature, mainly from the UK and Europe	1829.1L, adults aged ≥19 y	−0.07 kg CO_2_-eq/d				Diet quality was assessed by the Healthy Diet Indicator, MDS, and Dietary Approaches to Stop Hypertension score. Assessed by 2 dietary indexes and after adjustments for energy, MD was inversely associated with diet-related GHGE
Blackstone, 2018 [92], Modeling, United States	United States Dietary Guidelines Advisory Committee, 2015 [93]	Food Intakes Converted to Retail Commodities Database (FICRCD) [94], and LCA dataset	2000.00, not specified	24.70 kg CO_2_-eq/wk	397.00 kg carbon deficit/wk	Water depletion: 0.75 m^3^ water-eq/wk		The GHGE of MD were 0.4% lower than the healthy United States diet and 48.8% higher than VEG. LU of MD was 1.5% lower than healthy diet and 42.9% higher than VEG. MD had 6.7% and 16% more WF than healthy diet and VEG, respectively
Naja, 2018 [53], Cross-sectional study, Lebanon	A posteriori DP assessment: Lebanese-MD- Naja et al., 2015 [95]	Review of LCA	1000.00, adults aged > 18 y	0.38 (0.14–0.62) kg CO_2_-eq/d		243.35 (131.35–355.35) L/d	4.60 (1.73–7.47) MJ/d	The Lebanese-MD had a lower WF and GHGE than Lebanese adult, Western, and high-protein diets
Blas, 2019 [96], Modeling and data analysis, Spain	Bach-Faig et al., 2011 [26]	Mekonnen and Hoekstra, 2011 [51], 2012 [84]	1860.00, not specified			2538.00 L/person-day		Green WF was 39% higher for CurrentDiet than MD. Blue WF of CurrentDiet was 34% higher than MD. Grey WF of CurrentDiet was 48% higher than MD. This is relevant for semiarid countries, like the Mediterranean climate
Batlle-Bayer, 2019 [97], Modeling and data analysis, Spain	Bach-Faig et al., 2011 [26].	Life cycle inventories and LCA data	2383.00, adults	1.30 tCO_2_-eq/capita-year				The GHGE of the Spanish citizen diet (2006–2016) were 17% and 11% higher than NAOS and MD, respectively. When GHGE of the 3 food baskets were adjusted for nutritional score, GHGE were reduced by 42% and 35% for NAOS and MD, respectively
Naja, 2019, [98], Longitudinal observational study, Lebanon, Greece, Spain, France	Adh MD: Lebanese-MD (posteriori): variant of the MD- Naja et al., 2015 [95], aMED (Greece, priori): Fung et al., 2009 [99], rMED (Spain, priori): Buckland et al., 2009 [100], Med-DQI, [101], (France, priori): Trichopoulou et al., 2003 [73]	Review of existing LCAs	1000.00, adults (20 y of age or older)	–0.02; –0.02; –0.03; –0.06 kg CO_2_-eq/d		–0.66; –6.48; –14.34; –34.69 L/d	–0.07; 0.13; –0.00; 0.19 MJ/d	An inverse association between adh MD and WF and GHGE were found. No association was found with energy. Low adh MD among Lebanese adults was observed
Chapa, 2020 [102], Modeling and data analysis, United States	The sample 2-wk menu: USDA ChooseMyPlate guide and the work of Migala, 2019 [103]	LCA data ecoinvent v3.4 database: Wernet et al., 2016 [104], and LCA literature	2000.00, adults	6.26 (6.16–6.36) kg CO_2_-eq/kg food or 2.95 kg CO_2_-eq/kg food				MD had lower GHGE than the recommended healthy United States and more than recommended lacto-ovo vegetarian and “typical” United States dietary pattern
Grosso, 2020 [41], Cross-sectional study, Italy	Adh MD (priori): MDS: Trichopoulou et al., 2003 [73]	Sources of secondary data (LCA)	Adults	–0.01 (0.01)	–0.02 (0.01)	–6.41 (5664.00)	–0.04 (0.03)	Higher adherence to MD and Alternate Diet Quality Index was associated with lower GHGE. Higher adherence to Dietary Quality Index-International was associated with lower LU. Higher adherence to Nordic diet with lower LU and WF
González-García, 2020 [8], Modeling, Spain	Bach-Faig et al., 2011 [26]	LCA studies and WF data from Mekonnen and Hoekstra, 2011 [51], 2012 [84]	2228.00, adults	2.79 kg CO_2_-eq/person-day		3047.00 L/person-day		MD had 22.9% and 14.4% lower GHGE than SEAD and NAOS, respectively. MD had 18.9% and 11.4% lower WF than SEAD and NAOS, respectively
Rosi, 2020 [105], Longitudinal observational study, Italy	Adh MD (priori): MDS: Trichopoulou et al., 2003 [73], and adapted from Aparicio-Ugarriza et al., 2019 (HELENA study) [106]	Database Barilla Centre for Food and Nutrition, [107]	1000.00, children 8–10 y	Winter: 1480.00 (1190.00–1770.00) g CO_2_-eq/person-day; spring: 1532.00 (1206.00–1858.00) g CO_2_-eq/person-day	Winter: 10.20 (8.10–11.90)m[^2^]/person-day; spring: 10.10 (8.10–12.10-) m^2^/person-day			A small positive correlation was observed between adherence to MD and total GHGE and LU
Belgacem, 2021 [30], Modeling and data analysis, European Union	Dietary scenario: Davis et al., 2015 [108] adjusted Sinkko et al., 2019 [109]	Meta-analysis of food system impact studies to date: related LCA studies	2000.00, not specified	4.88 kg CO_2_-eq/person-day	14.80 m^2^/person-day	1079.96 L/person-day		Respectively, MD had 46.3% and 35.7% lower GHGE than WD and European diet; 55.4% and 41.4% lower LU than WD and European diet; 18.1% and 2.3% lower WF than European and WD
Telleria-Aramburu, 2021 [110], Cross-sectional study, Spain	Adh MD (priori): MDS: Panagiotakos et al., 2006 [111]	Literature review GHGE	1000.00, students 18–28 y	–0.02 kg CO–-eq/d				The low GHGE diets were consumed by subjects with low Healthy Eating Index-2010 scores and high MDS after controlling for sex, socioeconomic status, and body fat status
Vanham, 2021 [112], Modeling and data analysis, different countries	Bach-Faig et al., 2011 [26]	Mekonnen and Hoekstra, 2011 [51], 2012 [84]	M: 2500.00; W: 2000.00, (20–64 y)			2966.00^14^; 2818.00^15^; 2874.00^16^; 2571.00^17^; 2819.00^18^; 2819.00^19^; 4516.00^20^; 3946.00^21^; 4650.00^22^ l/person-day		The EAT-Lancet diet reduced the WF for all nations within the range 17%–48%. MD reduced the WF of the European countries, Turkey, Egypt, and Morocco within the range of 4%–35%. The EAT-Lancet diet had a lower WF than MD, however MD is probably more culturally accepted
Cambeses-Franco, 2022 [113], Modeling and data analysis, Spain	González-García et al., 2020 [8]	CF: literature. WF: first 2 steps of the 4-phase of the WFA methodology (WFN, 2020)	2228.00, Spanish W: middle-aged	2.21 kg CO_2_-eq/person-day		2826.00 L/person-day		IDG and MD provided the best profiles from a GHGE perspective. NAOS and MD showed higher WFs than IDG, DDG, and DGA. When compared with DDG and DGA, the nutritional value of MD was demonstrated with the highest nutrient-rich index and health gain score
Paris, 2022 [114], Modeling and data analysis, Germany	Bach-Faig et al., 2011 [26]. Fidanza, Alberti, 2005 [115]	Life cycle impact assessment: software Optimeal: Broekema et al., 2019 [116]	W: 1800.00–1830.00; M: 2230.00–2280.00; adults	M: 5.53 kg CO_2_-eq/person-day; W: 3.94 kg CO_2_-eq/person-day	M: 4.77 m^2^/person-day; W: 3.59 m^2^/person-day	M: 0.29 m^3^/d; W: 0.25 m^3^/d		DGE and MD decreased GHGE by 20%–30% and was >50% lower in the VD than RD. WF was 28% and 80% larger for MD and VD, respectively, than RD and DGE. MD had 20.4% and 1.1% lower LU than RD and DGE respectively, and 12.6% higher LU than VD
Naja, 2022 [117], Cross-sectional study, United Arab Emirates	Adh MD (priori): c-MED index	Review of LCAs analyses	1000.00, W: 19–50 y	−0.93 kg CO_2_-eq/kg of food consumed		−531.20 L/kg of food consumed	–1.27 MJ/kg food consumed	After adjustment for age, energy intake, and potential confounders, adherence to MD was associated with a 38%, 42.3%, and 7.1% decrease in GHGE, WF, and energy use, respectively
Castaldi, 2022 [118], Modeling and data analysis, Italy	Willet et al., 2019 [119]	Su-Eatable LIFE (SEL) database – Peterson et al. 2021 [[120], [121]]	2380.00, not specified	2.49 kg CO_2_-eq/person-day				MD had 44.2% and 38.2% GHGE lower than DP of 7 Mediterranean countries (Cyprus, Croatia, Greece, Italy, Portugal, Spain, and Malta) and the other 21 countries in the EU28 in 2017
Tepper, 2022, [35], Cross-sectional study, Israel	Adh MD (priori): Trichopoulou et al., 2003 [73]	LU- FAOstat data; WF- virtual water database; CF- LCAs studies review and relevant data from domestically grown commodities	Young adult participants aged 20–66 y	1.80 kg/CO_2_	3.70 m^2^/kg	411.20 L/person-day		MDS had 14.3% lower GHGE than SHED Index and the same as the EAT-Lancet Score. MDS LU was 11.9% lower than SHED Index and 8.1% higher than EAT-Lancet Score. MDS WF was 13.5% and 6.1% higher than EAT-Lancet Score and SHED Index, respectively

Abbreviations: Adh MD, adherence MD; aMED, alternate MD; CF, carbon footprint; c-MED, composite Mediterranean; DDG, Dutch Dietary Guidelines; DGA, American Dietary Guidelines; DGE, German Nutrition Society; DP, dietary pattern; F, female; GHGE, Greenhouse gas emissions; IDG, Italian Dietary Guidelines; LCA, Life Cycle Assessment; LU, land use; M, male; MD, Mediterranean Diet; MDS, MD Score; MEDQQI, MD Quality Index; mMDS, modified MDS; NAOS, Strategy for Nutrition, Physical Activity and Prevention of Obesity; RD, Reference Diet; rMED, Relative MD; SEAD, Southern European Atlantic diet; SHED, Sustainable Healthy Diet; VD, vegan diet; VEG, vegetarian diet; WF, water footprint; WFA, Water Footprint Assessment; WFN, Water Footprint Network; WD, Western diet.

1 Dubrovnik; 2 Lyon; 3 Athens; 4 Jerusalem; 5 Genoa; 6 Pisa; 7 Bologna; 8 Reggio Emilia; 9 Ljubljan; 10 Manresa; 11 Zaragoza;12 Ankara; 13 Istanbul; 14 Spain; 15 France; 16 Greece; 17 Italy; 18 Turkey; 19 Egypt; 20 Morocco; 21 Algeria; 22 Tunisia.

The selected articles included 25 modeling studies and ten epidemiological studies, 7 using cross-sectional and 3 using longitudinal designs. All were published after 2011. In the modeling studies, MD referred to Mediterranean models based on dietary recommendations or guidelines such as Bach-Faig et al. [26] and Trichopoulou et al. [73] (see Supplemental Table 1). In cross-sectional and longitudinal studies, the intake of the study population was assessed, and level of adherence to DP then evaluated with a priori or posteriori tools (see Supplemental Table 11).

To assess environmental sustainability, 24 studies compared MD with other DPs: European DP [[30], [57]], Western diet (WD) [[30], [47], [53], [60]], New Zealand DP, Asian style diet [58], Italian DP [[60], [62]], hyperproteic diet [[53], [62]], Dutch diet [[67], [77], [113]], semivegetarian diet [67], vegetarian diet (VEG) [[67], [71], [72], [74], [85], [92], [102]], vegan diet (VD) [[67], [86], [114]], omnivorous diet [76, pescetarian diet [[76], [85]], healthy diet [[74], [92]], LowLand Diet [77], Nordic diet [[41], [77], [87], [113]], American diet [[83], [113]], Strategy for Nutrition, Physical Activity and Prevention of Obesity (NAOS Strategy) [[8], [97], [113]], healthy United States and typical United States diet [102], Atlantic diet [8], EAT-Lancet diet [112], Italian Dietary Guidelines [113], and German diet [114]. An article modeled diets with an a posteriori approach, taking GHGE and the WF into account [80]. Four studies assessed environmental indicators alongside other dietary scores such as the Dietary Approach to Stop Hypertension [[41], [92]], the Healthy Diet Indicator [[89], [110]], the Alternate Diet Quality Index, the Dietary Quality Index-International [41], the EAT-Lancet Score, and the Sustainable Healthy Diet index [35]. Among the DPs analyzed, the percentage of diets that were most frequently compared with the MD were 60% for the current diets of the population under study, 22.8% for VEG, 20% for WD, 11.4% for Nordic diet, and 8.6% for VD.

The sustainability of MD was assessed in 21 studies by comparing dietary consumption data obtained from food balance sheets, daily note-taking on household food shopping, dietary records, national surveys or baskets, and/or food frequency questionnaires [[30], [35], [41], [47], [53], [57], [60], [62], [67], [71], [74], [85], [88], [89], [96], [97], [98], [105], [110], [112], [117]]. Of these, 11 also performed dietary scenarios and/or compared MD with other diets [[30], [47], [57], [60], [62], [67], [71], [74], [85], [97], [112]]. Using various indexes, adherence to MD was measured a priori in 7 articles [[35], [41], [88], [89], [105], [110], [117]] and a posteriori in 2 [[53], [98]] (Supplemental Table 11).

Diverse methods were employed to calculate environmental indicators, including LCA sources, secondary data, a European Environmentally Extended Input-Output model, and a hybrid input-output analysis. Data made available on daily caloric intake and the study population are presented in Table 1. Although 7 studies established a daily energy value for MD of 2000 kcal [[30], [47], [62], [67], [76], [92], [102]], other totals were stated, including 3483 [57], 2817.4 [58], 3649 [60], 2503 [77], and 1829.1 [89]. The study populations of 2 articles were exclusively male [[58], [77]] and 4 articles exclusively female [[67], [86], [113], [117]], whereas 10 included both [[8], [41], [47], [53], [57], [60], [89], [97], [98], [102]] and 13 did not specify [[30], [35], [62], [71], [72], [74], [76], [83], [85], [87], [88], [92], [96]]. Only 2 studies defined a different daily energy value for men and women [[112], [114]]. Two articles referred to children [[80], [105]] and 1 to university students [110]. Twelve specified age [[35], [53], [67], [77], [80], [89], [98], [105], [110], [112], [113], [117]], 10 referred to adults [[8], [41], [47], [57], [58], [60], [86], [97], [102], [114]], and 13 did not specify [[30], [62], [71], [72], [74], [76], [83], [85], [87], [88], [92], [96], [118]], although 1 provided the mean age of the study population [88].

Only 17% (*n* = 6) of the total articles included in the systematic review assessed both the environmental and the health aspect of the MD. The nutritional quality of the MD was analyzed using various indexes such as health score, nutrient-rich food index, nutritional quality index, nutrient-rich diet, and human health indicators. These studies evidence the health benefits of the MD [[67], [77], [86], [102], [113], [114]]. The studies that used the health score ranged between 122 and 178 points and between 13.51 and 90.6 points on the nutrient-rich food index, showing high nutritional quality (see Supplemental Table 12).

A total of 29 studies assessed GHGE, 11 articles assessed LU, 20 assessed WF, and 7 assessed energy use (see Table 2). A single environmental measure was addressed in 17 studies, of which 11 assessed only GHGE [[57], [58], [72], [76], [86], [87], [89], [97], [102], [110], [118]], the most commonly measured component, and 6 only analyzed WF [[60], [71], [83], [85], [96], [112]]. System boundaries were identified in studies assessing the environmental indicators of MD. Indicator calculation methods varied between studies and were also dependent on the stage of food production, consumption, and waste disposal (see Supplemental Table 13). Eleven studies evaluated environmental sustainability from cradle to farm, 5 from cradle to manufacture gates, 6 from cradle to retail, 9 from cradle to consumer, 2 from cradle to waste, and 2 did not specify. Moreover, diverse functional units were used to present results of the same environmental indicator with different units of mass, volume, energy, and time (see Supplemental Table 14). WF were measured taking into account different types of water. One study analyzed water depletion, defined as water use related to local water scarcity [92]. From a total of 19 articles, 6 analyzed the 3 types of WF (blue, green, and grey water), another six 2 types (blue and green), 1 assessed only 1 type of water, blue water (see Table 2). The other 6 studies did not specify the type; however, 4 of them used the term water consumption [[47], [80], [88], [114]], 1 article used different terms for referring to the WF [41], and the last used the term freshwater withdrawals [30].

**TABLE 2:** Environmental indicators of the articles included.

Article	Environmental indicator			
	GHGE	Land use	Water footprint	Others
Tukker, 2011 [57]	X			
Sáez-Almendros, 2013 [47]	X	X	X (water consumption)	Energy use
Wilson, 2013 [58]	X			
Capone, 2013 [60]			X (blue + green + grey)	
Germani, 2014 [62]	X	X	X (blue + green + grey)	
Van Dooren, 2014 [67]	X	X		
Vanham, 2014 [71]			X (blue + green + grey)	
Tilman, 2014 [72]	X			
Pairotti, 2014 [74]	X			Energy use
Vidal, 2015 [76]	X			
Van Dooren, 2016 [77]	X	X		
Benvenuti, 2016 [80]	X		X (water consumption)	
Blas, 2016 [83]			X (blue + green + grey)	
Vanham, 2016 [85]			X (blue + green)	
Castañé, 2017 [86]	X			
Ulaszewska, 2017 [87]	X			
Fresán, 2018 [88]	X	X	X (water consumption)	Energy use
Murakami, 2018 [89]	X			
Blackstone, 2018 [92]	X	X		Water depletion
Naja, 2018 [53]	X		X (blue + green)	Energy use
Blas, 2019 [96]			X (blue + green + grey)	
Batlle-Bayer, 2019 [97]	X			
Naja, 2019 [98]	X		X (blue + green)	Energy use
Chapa, 2020 [02]	X			
Grosso, 2020 [41]	X	X	X (NA)	Energy use
González-García, 2020 [8]	X		X (blue + green)	
Rosi, 2020 [105]	X	X		
Belgacem, 2021 [30]	X	X	X (freshwater withdrawals)	
Telleria-Aramburu, 2021 [110]	X			
Vanham, 2021 [112]			X (blue + green)	
Cambeses-Franco, 2022 [113]	X		X (blue + green + grey)	
Paris, 2022 [114]	X	X	X (water consumption)	
Naja, 2022 [17]	X		X (blue + green)	Energy use
Castaldi, 2022 [118]	X			
Tepper, 2022 [35]	X	X	X (blue)	
Total	29	11	19	7 (energy use)

Abbreviation: GHGE, Greenhouse gas emission.

The studies used different units to measure GHGE, LU, WF, and energy use. For example, the results of GHGE were presented in tons, kilograms, gigagrams, grams of CO_2_-eq per person, and per kilogram of food by day, week, month, and year. For WF, results were presented in tons, liters, cubic meters, and kilometers per person, as well as by day, week, month, and year. One study did not specify the units of measurement for environmental footprints used in the table of results [41]. Another measured environmental footprints per kilogram of food consumed [117]. Therefore, a formal meta-analysis was not undertaken due to this heterogeneity.

The environmental footprint of each food or food group within the MD is presented in Supplemental Table 15, ordered from highest to lowest based on available data or other relevant factors (justifications provided below Supplemental Table 15). The measurements are expressed in different units, including the quantity of each food, so direct comparability between articles is not feasible. Consequently, the food with the highest environmental footprint varies—sometimes it is meat or dairy, but in some cases, fruits or vegetables take the lead. This further underscores the heterogeneity we mention above. We could include 43% of the total articles in our systematic review in this table. Of these, 11 analyzed the GHGE, 8 the WF, and 2 the LU. For the GHGE, dairy products were the highest in 5 articles (45%) [[47], [86], [87], [97], [113]], followed by meat in 4 cases (36%) [[76], [92], [110], [118]], and a couple articles with fruits and/or vegetables at the top of the list [[53], [58]]. Oils were found to have the highest WF in 3 studies [[47], [96], [113]]. Both olive oil and a miscellaneous category (rest of food) rank first in terms of WF [83]. Meat was at the top of the list in a couple studies [[60], [67]], and fruit [92] and dairy [53] each appeared once. Finally, the highest food in LU was protein [92] and dairy products [47], according to each of the articles included in the table.

From the main results of each article included in our systematic review, 32 found MD to be a sustainable DP compared with other diets and/or scenarios [[30], [47], [57], [60], [62], [67], [76], [80], [83], [96], [105], [118]] or measured adherence [[35], [41], [53], [88], [89], [98], [110], [117]]. However, 17 studies [[58], [71], [72], [74], [77], [85], [86], [87], [92], [97], [102], [105], [112], [113], [114]] found that MD had more environmental impact in some variables, especially WF [[35], [41], [71], [85], [112], [113]], when compared with diets including less or no animal protein (for example, VD, VEG, semi-VEG, pescetarian, or pesco-VEG diet) or diets specifically designed to minimize environmental impact (for example, EAT-Lancet diet or NAOS Strategy). However, these studies did not take cultural factors into account. Some diets, such as the healthy United States diet and the Italian Guideline Diet [[93], [114]], had a lower WF than MD in the country of origin due to production systems and climate. Two other diets had a similar environmental impact to MD: the Nordic Diet and the German Diet [[77], [87], [114]].

**FIGURE 2:**
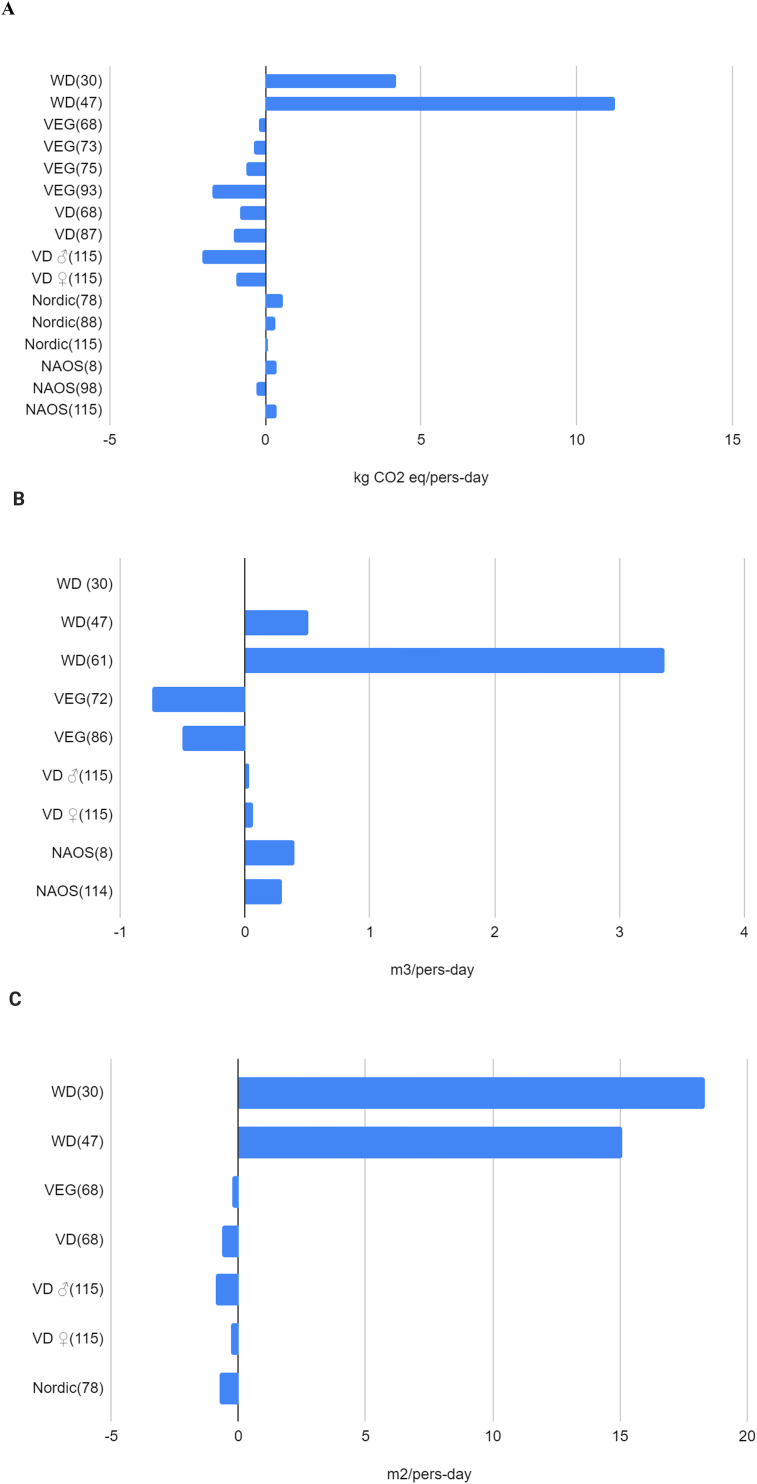
(A) Comparison of the greenhouse gas emissions of the main diets in this review with MD. Numbers between parentheses reference articles from which data has been obtained. Being Y axis = 0 the Mediterranean diet; NAOS, Strategy for Nutrition, Physical Activity and Prevention of Obesity; Nordic, Nordic diet; VD, vegan diet; VEG, vegetarian diet; WD: Western diet. Location belonging to the references: 8, Spain; 30, European Union; 47, Spain; 68, the Netherlands; 73, United States; 75, Italy; 78, the Netherlands; 88, Italy; 93, United States; 98, Spain; 115, Germany. Information about calculations and detailed numbers can be consulted in Table 3. (B) Comparison of the water footprints of the main diets in this review with MD. Numbers between parentheses reference articles from which data has been obtained. Being Y axis = 0 the Mediterranean diet; NAOS, Strategy for Nutrition, Physical Activity and Prevention of Obesity; VD, vegan diet; VEG, vegetarian diet; WD: Western diet. Location belonging to the references: 8, Spain; 30, European Union; 47, Spain; 61, Italy; 72, Italy; 86, European cities; 114, Spain; 115, Germany. Information about calculations and detailed numbers can be consulted in Table 4. (C) Comparison of the land use of the main diets in this review with MD. Numbers between parentheses reference articles from which data has been obtained. Being Y axis = 0 the Mediterranean diet; Nordic, Nordic diet; VD, vegan diet; VEG, vegetarian diet; WD: Western diet. Location belonging to the references: 30, European Union; 47, Spain; 68, the Netherlands; 78, the Netherlands; 115, Germany. Information about calculations and detailed numbers can be consulted in Table 5. MD, Mediterranean diet; VD, vegan diet; VEG, vegetarian diet; WD, western diet.

Comparisons between the MD and other DPs yielded varied results, with calculated ranges, when possible, of 1.03–5.08 kg CO_2_-eq/person-day for GHGE, 257.2–2735.2 L/person-day for WF, and 4–14.8 m^2^/person-day and 2.85–3.32 m^2^∗y/d for LU, over 2000 kcal. As the units of measurement were too diverse to be amalgamated, it was not possible to calculate any range for energy use. Because of the differences in each study, including the definition of the MD, the source of the environmental footprint data, and the stage of food production or the type of water included in the analysis, the wide range of these results makes comparisons between them impossible. Using the results for environmental footprints in the same measurement units, we created some figures (Figure 2A–C) and some tables ([TABLE 3, TABLE 4, TABLE 5]) to quantitatively compare the footprint of the most frequently assessed diets to the MD. To compare the environmental impact of each type of diet, we subtracted the environmental footprint corresponding to the specific diet from that of the MD and then plotted the differences: positive values when the other diet had a higher environmental impact or negative values when it had a lower environmental impact. For instance, GHGE in Van Dooren 2014 [67], MD has 3.41 kg CO_2_-eq/person-day compared with VEG diet with 3.2 kg CO_2_-eq/person-day, so when compared, VEG diet is −0.21 less GHGE.

**TABLE 3:** Greenhouse gas emissions in kg CO_2_ eq/person-day from different diets of the articles included.

First author, year, and location	MD	WD	VEG	VD	Nordic	NAOS	Equivalent results with MD = 0 kg CO_2_ eq/person-day
Pairotti, 2014 [74], Italy	12.33		11.7				VEG: –0.63
Belgacem, 2021 [30], European Union	4.88	9.08					WD: 4.2
Paris, 2022 [114], Germany	M: 5.53 W: 3.94			M: 3.5 W: 3			VD: M: –2.03, W: –0.94
Batlle-Bayer, 2019 [97], Spain	3.56					3.29	NAOS: –0.27
Blackstone, 2018 [92], United States	3.53		1.81				VEG: –1.72
Van Dooren, 2014 [67], the Netherlands	3.41		3.2	2.65			VEG: –0.21, VD: –0.8
Ulaszewska, 2017 [87], Italy	3.36				3.69		Nordic: 0.33
Van Dooren, 2016 [77], the Netherlands	3.24				3.82		Nordic: 0.58
Castañé, 2017 [86], Mediterranean Area	2.86			1.86			VD: –1
González-García, 2020 [8], Spain	2.79					3.15	NAOS: 0.36
Cambeses-Franco, 2022 [113], Spain	2.21				2.29	2.57	Nordic: 0.08, NAOS: 0.36
Sáez-Almendros, 2013 [47], Spain	2.19	13.42					WD: 11.23
Tilman, 2014 [72], United States	0.85		0.5				VEG: –0.35

Abbreviations: M, male; MD, Mediterranean diet; NAOS, strategy for nutrition, physical activity and prevention of obesity; Nordic, Nordic diet; VD, vegan diet; VEG, vegetarian diet; W, woman; WD, Western diet.

**TABLE 4:** Water footprint in m^3^/person-day from different diets of the articles included.

First author, year, and location	MD	WD	VEG	VD	NAOS	Equivalent results with MD = 0 m^3^/person-day
Vanham, 2014 [71], Italy	4.34		3.6			VEG: –0.74
Vanham, 2016 [85], European cities	2.64–3.65		2.1–3.2			VEG: –0,54 to –0,45
González-García, 2020 [8], Spain	3				3.4	NAOS: 0.4
Cambeses-Franco, 2022 [113], Spain	2.83				3.12	NAOS: 0.29
Capone, 2013 [60], Italy	2.64	6				WD: 3.36
Belgacem, 2021 [30], European Union	1.1	1.1				WD: 0
Sáez-Almendros, 2013 [47], Spain	0.82	1.33				WD: 0.51
Paris, 2022 [114], Germany	M: 0.29, W: 0.25			M: 0.32, W: 0.31		VD: M: 0.03, W: 0.06

Abbreviations: M, male; MD, Mediterranean diet; NAOS, strategy for nutrition, physical activity and prevention of obesity; VD, vegan diet; VEG, vegetarian diet; W, woman; WD, Western diet.

**TABLE 5:** Land use in m^2^/person-day from different diets of the articles included.

First author, year, and location	MD	WD	VEG	VD	Nordic	Equivalent results with MD = 0 m^2^/person-day
Belgacem, 2021 [30], European Union	14.8	33.15				WD: 18.35
Sáez-Almendros, 2013 [47], Spain	5.45	20.55				WD: 15.1
Paris, 2022 [114], Germany	M: 4.9 W: 3.6			M: 4 W: 3.3		VD: M: –0.9 W: –0.3
Van Dooren, 2016 [77], the Netherlands	4.15				3.42	Nordic: –0.73
Van Dooren, 2014 [67], the Netherlands	2.85		2.6	2.2		VEG: –0.25; VD: –0.65

Abbreviations: M, male; MD, Mediterranean diet; Nordic, Nordic diet; VD, vegan diet; VEG, vegetarian diet; W, woman; WD, Western diet.

As can be seen, WD scored significantly higher for the 3 indicators than MD. The Nordic diet scored higher for GHGE than MD, but slightly lower for LU. In 1 study, the NAOS diet scored slightly higher than MD for GHGE, but lower for the 2 other variables. WF obtained a higher result than MD in both studies in which it was addressed. VEG and VD scored lower for both GHGE and LU than MD, but VD scored higher for WF than MD.

### Quality assessment

Evaluation of the selected modeling studies using a modified critical appraisal checklist determined their quality to be high, with all but one receiving scores between 8 and 11 out of 12 (mean score of 9.5, see Supplemental Table 16). All studies stated the problem that prompted the study (Criterion 1). Of the 26 articles, 9 discussed the need for modeling compared with alternative methodologies (Criterion 2). Criterion 2 was included in the checklist to maintain consistency with previous reviews performed by the Dietary Guidelines Advisory Committee [55] and Nelson et al. [122]. However, because modeling is currently the only viable methodology for measuring the impact of diets on environmental outcomes and climate change, it may not warrant inclusion in future applications.

All selected studies described relevant factors and outcomes, as well as the models used (Criteria 3 and 4). Explanations of the rationale for the use of modeling were less common, although some articles provided this as strength of the methodology. When describing data sources, many studies cited literature reviews and/or analyses performed in previously published articles. This was usually in relation to LCA and WF analysis (Criterion 5). A discussion of assumptions within the analysis was completed by all but one study, with varying degrees of explanation. Supplemental materials often contained additional support for various assumptions (Criterion 6). All articles but one sufficiently discussed the base case analysis; however, the presentation of outcomes associated with the base case scenario varied (Criterion 7). Many studies examined the base case in the analysis, whereas others only calculated the impact of shifting current consumption to recommended diets. With the latter, it was possible to present results as a percent change to the recommended diet without outcomes reported on baseline conditions, although this only occurred in a few studies (Criterion 8).

There was consistent discussion of studies applying similar methodologies or examining similar research questions, as well as how the findings of this study were compared (Criterion 9). Eleven studies indicated the use of assumptions in the analysis to some degree; however, there were substantial disparities in discussions on how modeling assumptions might affect results, including the direction and magnitude of bias (Criterion 10). Various validation techniques were undertaken, including Monte Carlo simulations, providing upper and lower limits of estimates, and internal data consistency checks. All studies addressed settings in which results could be applied (Criterion 11). Only 3 studies indicated the need for future research and how future analyses could be improved (Criterion 12). Little attention was given to whether future improvements to the body of research could alter the results of the analysis at hand.

## Discussion

### Findings overview

This review advances our understanding by identifying and characterizing the environmental footprint of MD through assessments of its GHGE, WF, LU, and energy use. Among these indicators, GHGE (82.8% of the selected articles) and WF (57.1% of the selected articles) have received considerable attention in existing literature. The past decade has witnessed an upsurge in research on this topic.

This review found that 91% of studies considered MD to be a sustainable DP. The other 9% declared it to be less sustainable than other diets. Within the former, 40% featured another diet that was more sustainable than MD (for example, VD or VEG). All except one of the articles (25.7%) addressing adherence to MD reported an inverse correlation between such adherence and environmental footprints. Only 1 article indicated a direct correlation [105]. In the latter study, the greater quantity of food consumed by individuals who adhered more closely to the MD could potentially account for the higher environmental demands associated with their dietary choices. All articles that analyzed dietary consumption data or measured adherence, except the study discussed in the previous sentence, determined that MD reduced 1 or more environmental indicators compared with other diets. A recent study of nearly 30,000 French adults produced similar results: greater adherence to MD was associated with reductions of 15% in GHGE, 35% in LU, and 17% in energy use [123].

### Comparative analysis insights

Diets with animal food content (such as MD) are expected to result in higher environmental footprints in comparison to those with very little or none (that is, flexitarian, VEG, and VD) [7]. If food consumption is reduced only for environmental reasons, there is a risk of micronutrient and energy deficiencies [[67], [77], [86], [102], [113], [114]]. Following dietary guideline recommendations is generally associated with a lower environmental impact, but shifts toward healthier diets do not always result in lower footprints [[72], [124], [125], [126], [127], [128], [129]].

Mainly, MD has been shown to be a healthy diet [[5], [130], [131]]. Other diets considered in this review, which exhibited a lower environmental footprint, may have been found to be less healthy, as indicated by Tilman and Clark [72]. Likewise, a 2018 study found that MD exhibited a better rate of advancement period, with 3.10 y gained, than the provegetarian pattern, which produced a 1.2-y gain. Nevertheless, the provegetarian diet still attained the highest score for environmental benefits [132].

Fish protein is an important food supply for human consumption and is the main animal protein in MD [133]. As the articles in our review and the relevant literature make clear, animal and dairy products have the largest environmental footprint [[30], [92], [134], [135]]. It should be noted that some of the articles we reviewed lacked detailed data on this issue or, when specified, underestimated information. For instance, some studies that included wild fishery products only took aquaculture products (salmon, cod, and prawns, which represent 28%–33% of total fish and seafood) into account and stated a WF of zero [[8], [83], [96], [112], [113]]. The scarcity of information with which to estimate the WF of nonaquaculture fish species is a significant issue and brings these results into question. Given the choice of either making assumptions or excluding fish-related footprints, the authors of one study only included global averages of GHGE data and no other footprint coefficients. Using standardized methods according to best practices [136], the LCAs of the most commonly consumed fish products should be included in databases, such as Ecoinvent [92].

Fishing activity in the Mediterranean is mainly coastal and a high level of fishing activity has led to generalized overexploitation [137], especially in the European countries of Spain, France, and Italy, which account for almost 60% of fishery production [138]. Not only does overfish decrease biodiversity and erode natural resources [[139], [140]], unsustainable fishing techniques disrupt the marine environment and deplete the species we consume [88]. The consumption of fish 2 or 3 times a week is highly recommended from a health point of view [[141], [142], [143]], but this may be ecologically detrimental. It is necessary to ensure supplies originate from recognized sustainable sources and, in the case of wild-caught fish, from species that are not subjected to overfishing [88]. Therefore, the recommendation for fish consumption in MD has been modified to take sustainability into account [144]. Current advice is to include small fish from the Mediterranean Sea, such as anchovies, sardines, and mackerel, and reduce consumption of preferred species, such as tuna, salmon, and swordfish, which are subject to exploitation. In accordance with international public opinion [145], the French Agency for Food Safety [146], current average adequate consumption levels according to the ENALIA survey, prescribed levels of Ω-3, and environmental sustainability, the recommended frequency of fish consumption has been reduced from 3–4 times to 2–3 times a week [147]. Moreover, one of the articles we reviewed calculated that eliminating fish from MD and optimizing for emissions reduction could lower GHGE by 54% [58].

Global adoption of MD or a pescetarian diet by 2050 would require, respectively, a 62% or 188% increase in seafood production. If wild-caught landings remain at current levels, aquaculture, which expanded at 6.1%/y between 2002 and 2012 [148], would have to expand an additional 4.1%/y between 2010 and 2050 to meet the demands of a pescetarian diet [72]. Consumption of fish from aquaculture is recommended to preserve wild resources, reduce hunger, and improve nutrition. Directive 13 of the Italian Dietary Guidelines advises against disparaging the consumption of fish from aquaculture as a strategy to preserve wild resources, protect health through the higher quality of farmed fish, and protect the environment. Nevertheless, fish from aquaculture systems have a similar impact to meat in nutrient enrichment potentials and other impact categories not directly related to GHGE [74]. Concerns remain about fish welfare, profitable production, and the increased mortality rate among farmed fish over recent years [149].

Current data show a decline in adherence to the MD in the Mediterranean area, which is increasingly eroded by the effects of globalization, the homogenization of lifestyles, loss of awareness and appreciation, and a lack of interest among younger generations in their own cultural food heritage. The MD pattern is losing ground to a more Westernized eating pattern. Urbanization, the globalization of food systems, and the homogenization of food behaviors are causing a shift toward more ultraprocessed, protein- and sugar-rich foods, a trend which has been contributing to obesity and noncommunicable diseases, as well as putting pressure on the planet’s ecological assets [[5], [150]]. As disposable incomes rise and the middle class expands globally, consumers are making significant changes in their diets, shifting from staples such as fruits and vegetables to more expensive food products (such as fish, meat, and dairy products) and toward more processed foods (which may contain high levels of fat, sugar, or salt) [151]. A change in national consumption patterns is one of the main reasons why Spain is currently not self-sufficient in food and feed (for livestock) as it was 50 y ago and is now a country with equal levels of net agricultural imports and domestic crop production (measured in terms of nitrogen soil content) [152]. Spain is one of the European countries in which most meat per person is consumed per year, second only to Austria [153].

When consumed in Mediterranean countries, MD is expected to have a lower environmental footprint. Various factors, such as the culture of the area and climate, must be considered when measuring the environmental impact of a diet [22]. The type of product (plant- or animal-based), its origin, and the climate in which it is produced can all considerably affect environmental footprints, particularly WF. Vahnam et al. [154] highlight that, because of their higher temperature and lower rainfall, the countries of Southern Europe experience more plant evapotranspiration and higher WFs per ton of certain products than countries located in Northern Europe [8]. This could explain why many of the reviewed studies found MD to have a higher WF than other diets [[34], [35], [41], [71], [85], [92], [112], [113], [114]].

The number of studies evaluating the environmental impacts of MD has significantly increased in recent years. We can compare our results with those of a systematic review in 2016 [46], which reported 8 results for GHGE, 5 for LU, and 4 for WF in relation to MD. Consequently, it can be observed that, over the past 6 y, studies on the GHGE of MD have increased by 237.5%, by 80% for LU, and by 400% for WF. When we calculate the total of those 3 environmental footprints, we observe an increase in studies of 229.4%. However, it is important to note that the systematic review published in 2016 was not focused on analyzing MD environmental footprints, which could imply a potential omission of relevant studies. Despite the rapid increase in the number of publications on environmental footprint indicators in recent years, there have been few attempts to integrate different measures of environmental impact into a coherent framework. A functional unit is defined as a representative reference measure of a system under study, to which all inputs and outputs can be related [155]. The choice of functional unit can impact evaluations of environmental performance [156], as well as findings and recommendations [[118], [157], [158]]. Having reviewed life-cycle analysis-based functional units [159], Heller et al. state that the basis for diet comparisons should be nutritional [160]. However, the value of a certain nutrient in a single product is not static and, because it depends on the dietary context and the overall diet, cannot be said to have an absolute nutritional value. Furthermore, the complexity of capturing the nutritional function of a single food product is high due to the diverse number of nutrients [156]. Therefore, it is not possible to compare environmental indicators due to the use of different units of measurement, as well as different methodologies and system boundaries, and influencing factors such as climate and area. To rank environmental footprints in our review, we were only able to calculate GHGE from 14 of 29 articles (48%), WF from 7 of 20 articles (35%), and LU from 2 and 2—using different units—out of 10 articles (40%). This issue arose in a similar, earlier systematic review [147]. Also, as an Italian study noted, the LCA method inevitably suffers from the omissions required to make the method applicable and can lead to underestimating total impact when applied to household consumption [74]. For this reason, they chose to assess the environmental footprint of MD using a hybrid method, which addresses the stages of food production and consumption through LCA and other methodologies, in this case input-output analysis.

Although environmental indicators are intended to assign a final value for a DP impact, the majority of studies in our review interpreted their results through comparison (for example, MD compared with VD). Thus, the use of standard units would be highly beneficial. Also, various methods could be used together when the objective is to compare an ideal MD scenario (from recommendations) with the actual dietary intake of individuals with a high rate of adherence to this diet (assessed by MD scores).

The variety of MD measures used in the studies we reviewed impacts the results for environmental indicators, because the number of components (nutrients, foods, or food groups), classification categories, measurement scales, statistical parameters (mean, median), and the contribution of each component (positive or negative) to the final score are dissimilar [[53], [88], [89], [96], [98], [105], [110], [117]]. The relationship between MD food composition, adherence score, and the environmental aspects of MD still requires further investigation. Most imperative is the need for consensus: the fact that the environmental indicator may even vary within the same country and from rural to urban areas (for example, own crops, own livestock, or purchase in supermarkets) must be considered.

Those articles that compared the most common diets with MD produced very different results from the rest of the literature related to the same diets and environmental indicators. Like our results, 2 systematic reviews showed a reduction in both GHGE and LU for VD and VEG when compared with MD [[161], [162]]. Another systematic review found a reduction in the LU indicator for both diets [163]. However, unlike our results, the latter review discovered that WF increased with VEG, and reduced with VD. Similar results for VD were found in other studies [[45], [164]]. In addition, VEG increased GHGE more than VD, reduced LU and, in one scenario, increased WF [45]. This study also included the Nordic diet, which reduced GHGE and LU, but less than MD. In general terms, we can state that being able to say a diet increases or reduces an environmental indicator depends on the diet with which it is being compared. Also, it is understandable that the results of these studies differ, because they depend on so many variables.

### Strengths and limitations

One limitation of this systematic review is that by focusing on only 4 environmental indicators, it does not take such factors as biodiversity, nitrogen, phosphorus, and eutrophication potential into account. Another limitation of this review was the subjectivity of the a priori methods used in some studies. Because of the broad scope of the gray literature that could be a source of evidence, this was not taken into account. Neither did we limit our investigation to a specific population.

We considered evaluating the possible publication bias of MD and sustainability with a funnel plot and Egger’s test. However, this would require sample sizes, which only feature in articles measuring MD adherence (*n* = 9). Furthermore, ≤7 different indexes were used to measure adherence (see Supplemental Table 2). Thus, it was not possible to perform a publication bias assessment.

This systematic review is the second to address the sustainability of MD. The earlier review searched for all indicators used to measure the sustainability of MD. However, we have focused specifically on the 4 environmental footprints of GHGE, WF, LU, and energy use, and compared the MD with other diets. We secured a higher number of eligible articles for all environmental indicators.

### Research prospects and applications

Future studies investigating the environmental impact of diets should consider all dimensions of dietary sustainability, and specifically should take other environmental aspects of MD into account, such as biodiversity, nitrogen, phosphorus, and eutrophication potential. To make reliable and valid comparisons, diets and/or dietary scenarios need to be based on modeled scenarios, use the same units of measurement, or be based on very specific areas with the same characteristics. Further consensus on data sources and measurement units would be highly beneficial. Special consideration should be given to nutritional quality, which ought to be evaluated using pertinent nutrient-based indicators. Additionally, cultural acceptability—a crucial yet frequently overlooked aspect of sustainability—should be considered. Also, as well as the way products are harvested, fished, and raised, it is important to detail their origin when measuring sustainability indicators. Finally, beyond assessing the environmental impact of diets and identifying more sustainable diets, strategies to promote and enable the adoption of such diets by consumers have yet to be addressed. An alternative approach would be to detail the environmental impact of a diet in specific areas, taking into account that, due to climate variability and the characteristics of the area in question, these data may change over time. The point in the food production process (from cradle to farm gate, from cradle to consumer, or from cradle to waste) from which we measure the product's environmental impact must also be considered.

### Conclusions

This review identified 35 eligible articles, of which 82.8% measured GHGE, 54.3% the WF, 31.4% the LU, and 20% the energy use. We have summarized the principal results from each study as a 91% confirmation of MD as a sustainable DP. MD exhibited higher environmental footprints than VEG and VD but lower environmental impacts than most present-day diets, including the United States Western DP. Both MD and the Nordic diet showed similar environmental footprints.

The reviewed studies analyze the environmental sustainability of the MD using one or more of the main and commonly used impact indicators (such as GHGE, WF, LU, and energy use). Furthermore, a limited number of studies also explore the dimension of healthy eating patterns by employing indexes that measure nutrient quality. Our article provides a summary of the environmental footprints associated with MD and elucidates them within the context of our food system, considering health, environmental factors, and potentially the socioeconomic dimension, which warrants further investigation.

According to the evidence, it is widely accepted that MD is a healthy DP. Diverse studies suggest that greater adherence to MD has a protective effect for noncommunicable diseases, especially those associated with age, and decreases the risk of overall mortality with better quality of life; it is found to be helpful in the prevention and treatment of cardiovascular and metabolic diseases, certain neurodegenerative disorders, and cancers, reduces the risk of fractures due to fragility, helps maintain cognitive function, and protects against depression. The study with the most evidence, the PREDIMED (prevention with MD) clinical trial, points to a 30% prevention of cardiovascular diseases. MD is effective in preventing obesity and metabolic syndrome in healthy or at-risk individuals; reduces the risk of mortality in people who are overweight or obese; decreases the incidence of type 2 diabetes mellitus and cardiovascular disease in healthy individuals; and reduces the severity of symptoms. With our results, we can also state that MD is sustainable and, therefore, is aligned with planetary health. This systematic review clarifies the importance of this diet and its benefits, and the need to promote health and educational policies that facilitate its adherence among Mediterranean populations that have abandoned their traditional DP, with the aim of protecting and safeguarding MD.

Only 20% of European dietary guidelines incorporate food sustainability. In Mediterranean countries where MD is one of the diet options seeking to minimize dietary environmental impact, food-based dietary guidelines should not only encompass the health aspect, as they traditionally have, but also include considerations for the environment. An example of this approach is evident in the food-based dietary guidelines of the Spanish Society of Community Nutrition, which endorse the traditional MD and promote a healthy, conscious lifestyle, by emphasizing the importance of allocating sufficient time for food selection, cooking, and eating together. Moreover, it encourages consumers to prioritize locally grown and seasonal foods, advocate for inquiries into food production practices, and demand sustainability throughout the entire food supply chain. This includes considerations for minimizing food waste, promoting recycling, and reducing packaging and associated waste. Additionally, there is an updated version of the MD food pyramid, which integrates the sustainability aspect [29].

As observed in this systematic review, there is a lack of consensus regarding the units of measurement and other factors to consider when analyzing the environmental impact of one or more DPs. It is important and relevant to consider the sustainable diet definition when measuring the sustainability of a diet or making comparisons between different DPs. Besides, future inclusive dialogs between scientists, policymakers, and other stakeholders, including various actors in the food system and beyond, are required to provide a resilient and sustainable food system for planetary health. Future policies require research and should account for political, social, and economic dimensions, as well as trade-offs, to holistically modify complex food systems [[22], [165], [166], [167], [168], [169], [170], [171]]. The promotion of a healthy and sustainable food model across the WHO European Region will not only benefit human health, but also planetary health.

## Author contributions

The authors’ responsibilities were as follows – VL-C, MB-P, AB-F: designed the research and drafted the manuscript, conducted the research, analyzed data, wrote the manuscript, and had primary responsibility for final content; MB-R, CO-G: reviewed and edited the manuscript; and all authors: read and approved the final manuscript.

## Conflict of interest

The authors report no conflicts of interest.

## Funding

The authors reported no funding received for this study.

## Data availability statement

▪
