# Supplementary material for: Environmental and Health Sustainability of the Mediterranean Diet: A Systematic Review
Source: Adv Nutr. 2024 Oct 18;15(12):100322. doi: 10.1016/j.advnut.2024.100322 (PMC11605453; doi:10.1016/j.advnut.2024.100322)
Supplement: multimedia component 1 [file mmc1.docx]

**Environmental and Health Sustainability of the Mediterranean Diet: A Systematic Review**

Victoria Lorca-Camara

**Supplementary Material (SM):**

**Supplementary Table SM1.** Composition of the Mediterranean diet in modeling studies

| **Reference** | **Composition of MD** |
| --- | --- |
| Tukker et al., 2011 | Fat: 34.5% of E; PROT: 11.8% of E; SFAt: 8.4% of E. |
| Bach-Faig et al., 2011 | Every Main Meal: F 1-2, v >/= 2s, variety of colors/textures (Cooked/Raw), olive oil, bread/pasta/rice/couscous/other cereals 1-2s (preferably whole grain). Every day: Dairy 2s (preferred low fat), olives/nuts/seeds 1-2s, herbs/spices/garlic/onions (less added salt), variety of flavors. Weekly: Potatoes </= 3s, white meat 2s, fish/seafood >/= 2s, eggs 2-4s, legumes >/= 2s, red meat < 2s, processed meat </= 1s, sweets </=2s. |
| Trichopoulou et al., 2009 | V: 549g (excluding starchy root crops: potatoes, taro and kumara); F and nuts: 363g; Fish and seafood: 24g; Olive oil: 56g; Salt in cooking: 0.5 teaspoon (3g). SFA: 13g, PUFA: 14g, PROT: 100g, Dietary fiber: 57g. |
| Willett et al., 2019 | G/d: Rice, wheat, corn, and other: 232g; Potatoes and cassava: 50g; v: 300g; f: 200g; Whole milk or derivative equivalents: 250g; Beef and lamb: 7g; Pork: 7g; Chicken and other poultry: 29g; Eggs: 13g; Fish: 28g; Legumes: 100g; Tree nuts: 25g; Palm oil: 6.8g; Unsaturated oils: 40g; Dairy fats (included in milk): 0g; Lard or tallow: 5g; Sweeteners: 31g. |
| Italian Institute of Food Science of the La Sapienza University-Rome, 2006 | Kg/capita/year: V 156.4, F 140.8, Milk 65.2, Bread 41.7, Yogurt 26.1, Meat 26.1, Pasta 25, Potato 20.9, F juice 19.5, Fish 15.6, Extra virgin olive oil 10.4, Rice 8.3, Meat cuts 7.8, Biscuits 7.3, Cheese 7.3, Eggs 6.3, Sugar 4.7, Legumes 3.1, Butter 2.6, Honey 0.8. |
| Del Balzo et al., 2012 | E 2000kcal/d: CHO 55–60% of E; PROT 10–12% of E; Fat 30% of E. |
| LARN, 2012 | G of F: 150 (3p/d); Seasonal v: 200 (2p/d); Nuts: 15 (3p/weekly); Pulses: 50 (2p/d); Pasta or rice: 80 (1 p/d); Bread: 50 (3p/d); Potatoes: 200 (2p/weekly); EVO oil: 10 (3p/d); Red meat: 100 (1p/weekly); White meat: 100 (2p/weekly); Processed meat: 50 (2p/weekly); Cheese fresh: 100 (2p/weekly); Eggs: 50 (2p/weekly); Fish: 150 (2p/weekly); Milk: 125 (1p/d); Yogurt: 125 (1p/d); Cookies: 30 (1p/d); Sugar: 5 (1p/d); Dessert: 100 (1p/weekly). |
| Dutch Dietary Guidelines: Health Council, 2006 | G of V: 300; Fresh: 300; F: 250; Bread: 210; Grains, potatoes, pulses: 200; Milk and milk products: 300; Cheese: 15; Meat, fish, eggs and meat substitutes: 100; Oils, fats, sauces: 45; Other: 45; Non-alcoholic drinks: 1500ml; Alcoholic drinks: 150ml; Other extra products: 200 kcal. |
| Modern Diet Mediterranean Food Pyramid, INRAN | Monthly portions/capita: Bread, pasta and flour products: 120; Potatoes, v, and f: 158; Meat and meat products: 14; Dairy products: 75; Fish: 8. |
| Diet published by Benidorm Clinical Hospital | G for Breakfast: Milk with coffee: 200cc; Sugar: 10; Bread: 60; Butter: 15; Marmalade: 50. G for Lunch: Rice/pasta: 100 or Potatoes: 300; Meat: 120 or Fish: 150 or 2 Eggs; V: 200; Bread: 60; F: 130. G for Snack: Milk: 200cc; Biscuits: 40; Sugar: 10. G for Dinner: Rice/pasta: 30 or Potatoes: 150; V/salad: 200; Meat: 120 or Fish: 150 or 2 Eggs; Bread: 60; F: 130. |
| Guide of health and nutritional practices: City of Rome, 2013 | G/Month: Minimizes GHGE- E: 544.52kcal; PROT: 24.13; Lipids: 21.92; CHO: 66.6; Fibers: 8.42; Sugars: 22.41; Sodium: 338.82mg. Minimizes WF- E: 541.66kcal; PROT: 24.23; Lipids: 20.55; CHO: 68.97; Fibers: 8.05; Sugars: 22.43; Sodium: 361.95mg. |
| INRAN guidelines, 2003 | Kg/week: Meat and cold cuts: 0.26; Poultry, fish, legumes and eggs: 0.68; Milk and dairy products: 2.83; Herbs, oils, condiments: 0.21; Cereals and potatoes: 2.85; F and V: 5.4; Water: 14.5. |
| Dietary Guidelines Advisory Committee, 2015 | Weekly: F: 17.5 cups; V: 17.5 cups; Grains: 42 oz; PROT: 45.5 oz; Dairy: 14 cups; Oils: 189 g. |
| USDA ChooseMyPlate guide and Migala, 2019 | On a table without specific numbering of the measures or functional unit: Broccoli, tomato, lentils, potato, mushrooms, eggplant, strawberries, grapes, orange, brown rice, pasta, yogurt, mozzarella cheese, sardines, salmon, chicken, almonds, olive oil, butter. |
| González-García et al., 2020 | G/p-d: F: 560; V: 700; Pulses: 20; Starch-based products: 315; Nuts and olives: 37.5; Dairy products: 270; Eggs: 24.86; Meat: 48.21; Fish and seafood: 39.29; Sweets: 6.43; Oils and fats: 45. |
| Davis et al., 2015 adjusted Sinkko et al., 2019 | G/p-d on a chart without specific numbering of the measures: Meat, fish, dairy products, cereal-based products, sugar, oils, tubers, v, legumes, f, nuts. |
| Fidanza F, Alberti A, 2005 | E%: Cereals: 48-52; Extra virgin olive oil: 14.5-16.6; V: 5-7; Pulses: 4.4-6.6; f 2-2.6; Fish: 1.6-2; Red wine: 4.2-6; Meat: 2.6-4; Milk and dairy products: 1.3-1.8; Eggs: 0.8-1.4; Animal fats: 1-2. |

CHO, Carbohydrates; d, day; E, Energy Intake; F, Fruit; g, grams; GHGE, Greenhouse gas emissions; INRAN, National Institute of Research on Food and Nutrition; kg, kilograms; mg, milligrams; NA, Data not available; p, person; PROT, Proteins; PUFA, Polyunsaturated fats; S, Servings; SFA, Saturated fatty acids; V, Vegetables; WF, Water Footprint.

**Supplementary Table SM2.** STROBE Statement Fresán U, 2017

| **Item Recommendation Reported on manuscript** | **Page** |
| --- | --- |
| **Title and abstract** |  |
| (a) Indicate the study’s design with a commonly used term in the title or the abstract | 1 |
| (b) Provide in the abstract an informative and balanced summary of what was done and what was found | 1 |
| **Introduction** |  |
| Background/rationale |  |
| Explain the scientific background and rationale for the investigation being reported | 1, 2 |
| Objectives |  |
| State specific objectives, including any pre-specified hypotheses | N |
| **Methods** |  |
| Study design |  |
| Present key elements of study design early in the paper | 2 |
| Setting |  |
| Describe the setting, locations, and relevant dates, including periods of recruitment, exposure, follow-up, and data collection | 2, 3 |
| Participants |  |
| (a) Cohort study—give the eligibility criteria, and the sources and methods of selection of participants. Describe methods of follow-up | 2 |
| Cross-sectional study—give the eligibility criteria, and the sources and methods of selection of participants | NA |
| (b) Cohort study—for matched studies, give matching criteria and number of exposed and unexposed | NA |
| Variables |  |
| Clearly define all outcomes, exposures, predictors, potential confounders, and effect modifiers. Give diagnostic criteria, if applicable | 2, 3, 8 |
| Data sources/ measurement |  |
| For each variable of interest, give sources of data and details of methods of assessment (measurement). Describe comparability of assessment methods if there is more than one group | 2, 3, supl. Material |
| Bias |  |
| Describe any efforts to address potential sources of bias | 2, 3 |
| Study size |  |
| Explain how the study size was arrived at | 2 |
| Quantitative variables |  |
| Explain how quantitative variables were handled in the analyses. If applicable, describe which groupings were chosen, and why | 2, 3 |
| Statistical methods |  |
| (a) Describe all statistical methods, including those used to control for confounding | 2, 3 |
| (b) Describe any methods used to examine subgroups and interactions | 2, 3 |
| (c) Explain how missing data were addressed | 2, 3, 8 |
| (d) Cohort study—if applicable, explain how loss to follow-up was addressed | 2 |
| Cross-sectional study—if applicable, describe analytical methods taking account of sampling strategy | NA |
| (e) Describe any sensitivity analyses | 3 |
| **Results** |  |
| Participants |  |
| (a) Report the numbers of individuals at each stage of the study—eg, numbers potentially eligible, examined for eligibility, confirmed eligible, included in the study, completing follow-up, and analyzed | 2 |
| (b) Give reasons for non-participation at each stage | 2 |
| (c) Consider use of a flow diagram | N |
| Descriptive data |  |
| (a) Give characteristics of study participants (eg, demographic, clinical, social) and information on exposures and potential confounders | 2, 3, 4 |
| (b) Indicate the number of participants with missing data for each variable of interest | 2 |
| (c) Cohort study—summarize follow-up time (eg, average and total amount) | 2 |
| Outcome data |  |
| Cohort study—report numbers of outcome events or summary measures over time | 2 |
| Cross-sectional study—report numbers of outcome events or summary measures | NA |
| Main results |  |
| (a) Give unadjusted estimates and, if applicable, confounder-adjusted estimates and their precision (eg, 95% confidence interval). Make clear which confounders were adjusted for and why they were included | 3, 4, 5 |
| (b) Report category boundaries when continuous variables were categorized | N |
| (c) If relevant, consider translating estimates of relative risk into absolute risk for a meaningful time period | N |
| Other analyses |  |
| Report other analyses done—eg, analyses of subgroups and interactions, and sensitivity analyses | 3, 4, 5 |
| **Discussion** |  |
| Key results |  |
| Summarize key results with reference to study objectives | 5, 6 |
| Limitations |  |
| Discuss limitations of the study, taking into account sources of potential bias or imprecision. Discuss both direction and magnitude of any potential bias | 8 |
| Interpretation |  |
| Give a cautious overall interpretation of results considering objectives, limitations, multiplicity of analyses, results from similar studies, and other relevant evidence | 6, 7, 8 |
| Generalisability |  |
| Discuss the generalisability (external validity) of the study results | 8 |
| **Other information** |  |
| Funding |  |
| Give the source of funding and the role of the funders for the present study and, if applicable, for the original study on which the present article is based | 8 |

N, No; NA, Not Available.

**Supplementary Table SM3.** STROBE Statement Murakami K, 2018

| **Item Recommendation Reported on manuscript** | **Page** |
| --- | --- |
| **Title and abstract** |  |
| (a) Indicate the study’s design with a commonly used term in the title or the abstract | 1 |
| (b) Provide in the abstract an informative and balanced summary of what was done and what was found | 1 |
| **Introduction** |  |
| Background/rationale |  |
| Explain the scientific background and rationale for the investigation being reported | 2 |
| Objectives |  |
| State specific objectives, including any pre specified hypotheses | N |
| **Methods** |  |
| Study design |  |
| Present key elements of study design early in the paper | 2 |
| Setting |  |
| Describe the setting, locations, and relevant dates, including periods of recruitment, exposure, follow-up, and data collection | 2, 3, 4 |
| Participants |  |
| (a) Cohort study—give the eligibility criteria, and the sources and methods of selection of participants. Describe methods of follow-up | NA |
| Cross-sectional study—give the eligibility criteria, and the sources and methods of selection of participants | 2, 3 |
| (b) Cohort study—for matched studies, give matching criteria and number of exposed and unexposed | NA |
| Variables |  |
| Clearly define all outcomes, exposures, predictors, potential confounders, and effect modifiers. Give diagnostic criteria, if applicable | 2, 3, 4 |
| Data sources/ measurement |  |
| For each variable of interest give sources of data and details of methods of assessment (measurement). Describe comparability of assessment methods if there is more than one group | 2, 3, 4 |
| Bias |  |
| Describe any efforts to address potential sources of bias | 4, 8 |
| Study size |  |
| Explain how the study size was arrived at | 2 |
| Quantitative variables |  |
| Explain how quantitative variables were handled in the analyses. If applicable, describe which groupings were chosen, and why | 2, 3, 4 |
| Statistical methods |  |
| (a) Describe all statistical methods, including those used to control for confounding | 2, 3, 4 |
| (b) Describe any methods used to examine subgroups and interactions | 2, 3, 4 |
| (c) Explain how missing data were addressed | 2, 4, 6, 7 |
| (d) Cohort study—if applicable, explain how loss to follow-up was addressed | NA |
| Cross-sectional study—if applicable, describe analytical methods taking account of sampling strategy | NA |
| (e) Describe any sensitivity analyses | 4 |
| **Results** |  |
| Participants |  |
| (a) Report the numbers of individuals at each stage of the study—eg, numbers potentially eligible, examined for eligibility, confirmed eligible, included in the study, completing follow-up, and analyzed | 2 |
| (b) Give reasons for non-participation at each stage | 2 |
| (c) Consider use of a flow diagram | N |
| Descriptive data |  |
| (a) Give characteristics of study participants (eg, demographic, clinical, social) and information on exposures and potential confounders | 2, 5 |
| (b) Indicate the number of participants with missing data for each variable of interest | 2 |
| (c) Cohort study—summarize follow-up time (eg, average and total amount) | NA |
| Outcome data |  |
| Cohort study—report numbers of outcome events or summary measures over time | NA |
| Cross-sectional study—report numbers of outcome events or summary measures | 4, 5, 6 |
| Main results |  |
| (a) Give unadjusted estimates and, if applicable, confounder-adjusted estimates and their precision (eg, 95% confidence interval). Make clear which confounders were adjusted for and why they were included | 4, 5, 6, 7 |
| (b) Report category boundaries when continuous variables were categorized | N |
| (c) If relevant, consider translating estimates of relative risk into absolute risk for a meaningful time period | NA |
| Other analyses |  |
| Report other analyses done—eg, analyses of subgroups and interactions, and sensitivity analyses | 5, 6, 7 |
| **Discussion** |  |
| Key results |  |
| Summarize key results with reference to study objectives | 6 |
| Limitations |  |
| Discuss limitations of the study, taking into account sources of potential bias or imprecision. Discuss both direction and magnitude of any potential bias | 7, 8 |
| Interpretation |  |
| Give a cautious overall interpretation of results considering objectives, limitations, multiplicity of analyses, results from similar studies, and other relevant evidence | 6, 7, 8 |
| Generalisability |  |
| Discuss the generalisability (external validity) of the study results | 6, 7, 8 |
| **Other information** |  |
| Funding |  |
| Give the source of funding and the role of the funders for the present study and, if applicable, for the original study on which the present article is based | 8 |

N=No; NA= Not Available.

**Supplementary Table SM4.** STROBE Statement Naja F, 2018

| **Item Recommendation Reported on manuscript** | **Page** |
| --- | --- |
| **Title and abstract** |  |
| (a) Indicate the study’s design with a commonly used term in the title or the abstract | 1 |
| (b) Provide in the abstract an informative and balanced summary of what was done and what was found | 1 |
| **Introduction** |  |
| Background/rationale |  |
| Explain the scientific background and rationale for the investigation being reported | 1, 2 |
| Objectives |  |
| State specific objectives, including any pre specified hypotheses | N |
| **Methods** |  |
| Study design |  |
| Present key elements of study design early in the paper | 2, 3 |
| Setting |  |
| Describe the setting, locations, and relevant dates, including periods of recruitment, exposure, follow-up, and data collection | 2, 3 |
| Participants |  |
| (a) Cohort study—give the eligibility criteria, and the sources and methods of selection of participants. Describe methods of follow-up | NA |
| Cross-sectional study—give the eligibility criteria, and the sources and methods of selection of participants | 2 |
| (b) Cohort study—for matched studies, give matching criteria and number of exposed and unexposed | NA |
| Variables |  |
| Clearly define all outcomes, exposures, predictors, potential confounders, and effect modifiers. Give diagnostic criteria, if applicable | 2, 3, 4, 5 |
| Data sources/ measurement |  |
| For each variable of interest give sources of data and details of methods of assessment (measurement). Describe comparability of assessment methods if there is more than one group | 2, 3, 4, 5 |
| Bias |  |
| Describe any efforts to address potential sources of bias | N |
| Study size |  |
| Explain how the study size was arrived at | 2 |
| Quantitative variables |  |
| Explain how quantitative variables were handled in the analyses. If applicable, describe which groupings were chosen, and why | 2, 3, 4, 5 |
| Statistical methods |  |
| (a) Describe all statistical methods, including those used to control for confounding | 2, 3, 4, 5 |
| (b) Describe any methods used to examine subgroups and interactions | 2, 3, 4 |
| (c) Explain how missing data were addressed | 2, 8, 9 |
| (d) Cohort study—if applicable, explain how loss to follow-up was addressed | NA |
| Cross-sectional study—if applicable, describe analytical methods taking account of sampling strategy | 2 |
| (e) Describe any sensitivity analyses | N |
| **Results** |  |
| Participants |  |
| (a) Report the numbers of individuals at each stage of the study—eg, numbers potentially eligible, examined for eligibility, confirmed eligible, included in the study, completing follow-up, and analyzed | 2 |
| (b) Give reasons for non-participation at each stage | 2 |
| (c) Consider use of a flow diagram | N |
| Descriptive data |  |
| (a) Give characteristics of study participants (eg, demographic, clinical, social) and information on exposures and potential confounders | 2, 5 |
| (b) Indicate the number of participants with missing data for each variable of interest | N |
| (c) Cohort study—summarize follow-up time (eg, average and total amount) | NA |
| Outcome data |  |
| Cohort study—report numbers of outcome events or summary measures over time | NA |
| Cross-sectional study—report numbers of outcome events or summary measures | 5, 6 |
| Main results |  |
| (a) Give unadjusted estimates and, if applicable, confounder-adjusted estimates and their precision (eg, 95% confidence interval). Make clear which confounders were adjusted for and why they were included | 5, 6 |
| (b) Report category boundaries when continuous variables were categorized | N |
| (c) If relevant, consider translating estimates of relative risk into absolute risk for a meaningful time period | NA |
| Other analyses |  |
| Report other analyses done—eg, analyses of subgroups and interactions, and sensitivity analyses | 5, 6 |
| **Discussion** |  |
| Key results |  |
| Summarize key results with reference to study objectives | 6 |
| Limitations |  |
| Discuss limitations of the study, taking into account sources of potential bias or imprecision. Discuss both direction and magnitude of any potential bias | 8, 9 |
| Interpretation |  |
| Give a cautious overall interpretation of results considering objectives, limitations, multiplicity of analyses, results from similar studies, and other relevant evidence | 6, 7, 8, 9 |
| Generalisability |  |
| Discuss the generalisability (external validity) of the study results | 8, 9 |
| **Other information** |  |
| Funding |  |
| Give the source of funding and the role of the funders for the present study and, if applicable, for the original study on which the present article is based | 9 |

N, No; NA, Not Available.

**Supplementary Table SM5.** STROBE Statement Naja F, 2019

| **Item Recommendation Reported on manuscript** | **Page** |
| --- | --- |
| **Title and abstract** |  |
| (a) Indicate the study’s design with a commonly used term in the title or the abstract | N |
| (b) Provide in the abstract an informative and balanced summary of what was done and what was found | 1 |
| **Introduction** |  |
| Background/rationale |  |
| Explain the scientific background and rationale for the investigation being reported | 1, 2 |
| Objectives |  |
| State specific objectives, including any pre specified hypotheses | N |
| **Methods** |  |
| Study design |  |
| Present key elements of study design early in the paper | 2, 3 |
| Setting |  |
| Describe the setting, locations, and relevant dates, including periods of recruitment, exposure, follow-up, and data collection | 2, 3 |
| Participants |  |
| (a) Cohort study—give the eligibility criteria, and the sources and methods of selection of participants. Describe methods of follow-up | NA |
| Cross-sectional study—give the eligibility criteria, and the sources and methods of selection of participants | 2, 3 |
| (b) Cohort study—for matched studies, give matching criteria and number of exposed and unexposed | NA |
| Variables |  |
| Clearly define all outcomes, exposures, predictors, potential confounders, and effect modifiers. Give diagnostic criteria, if applicable | 3, 4, 5 |
| Data sources/ measurement |  |
| For each variable of interest give sources of data and details of methods of assessment (measurement). Describe comparability of assessment methods if there is more than one group | 2, 3, 4, 5 |
| Bias |  |
| Describe any efforts to address potential sources of bias | N |
| Study size |  |
| Explain how the study size was arrived at | 2, 3 |
| Quantitative variables |  |
| Explain how quantitative variables were handled in the analyses. If applicable, describe which groupings were chosen, and why | 3, 4, 5 |
| Statistical methods |  |
| (a) Describe all statistical methods, including those used to control for confounding | N |
| (b) Describe any methods used to examine subgroups and interactions | 3, 4, 5 |
| (c) Explain how missing data were addressed | 2, 12 |
| (d) Cohort study—if applicable, explain how loss to follow-up was addressed | NA |
| Cross-sectional study—if applicable, describe analytical methods taking account of sampling strategy | 2 |
| (e) Describe any sensitivity analyses | 5 |
| **Results** |  |
| Participants |  |
| (a) Report the numbers of individuals at each stage of the study—eg, numbers potentially eligible, examined for eligibility, confirmed eligible, included in the study, completing follow-up, and analyzed | 2, 3 |
| (b) Give reasons for non-participation at each stage | 2 |
| (c) Consider use of a flow diagram | N |
| Descriptive data |  |
| (a) Give characteristics of study participants (eg, demographic, clinical, social) and information on exposures and potential confounders | 2, 5, 6 |
| (b) Indicate the number of participants with missing data for each variable of interest | N |
| (c) Cohort study—summarize follow-up time (eg, average and total amount) | NA |
| Outcome data |  |
| Cohort study—report numbers of outcome events or summary measures over time | NA |
| Cross-sectional study—report numbers of outcome events or summary measures | 5, 6, 7, 8, 9, 10 |
| Main results |  |
| (a) Give unadjusted estimates and, if applicable, confounder-adjusted estimates and their precision (eg, 95% confidence interval). Make clear which confounders were adjusted for and why they were included | 5, 6, 7, 8, 9, 10 |
| (b) Report category boundaries when continuous variables were categorized | N |
| (c) If relevant, consider translating estimates of relative risk into absolute risk for a meaningful time period | NA |
| Other analyses |  |
| Report other analyses done—eg, analyses of subgroups and interactions, and sensitivity analyses | 9, 10 |
| **Discussion** |  |
| Key results |  |
| Summarize key results with reference to study objectives | 10 |
| Limitations |  |
| Discuss limitations of the study, taking into account sources of potential bias or imprecision. Discuss both direction and magnitude of any potential bias | 12 |
| Interpretation |  |
| Give a cautious overall interpretation of results considering objectives, limitations, multiplicity of analyses, results from similar studies, and other relevant evidence | 10, 11, 12 |
| Generalisability |  |
| Discuss the generalisability (external validity) of the study results | 12 |
| **Other information** |  |
| Funding |  |
| Give the source of funding and the role of the funders for the present study and, if applicable, for the original study on which the present article is based | 13 |

N, No; NA, Not Available.

**Supplementary Table SM6.** STROBE Statement Grosso G, 2020

| **Item Recommendation Reported on manuscript** | **Page** |
| --- | --- |
| **Title and abstract** |  |
| (a) Indicate the study’s design with a commonly used term in the title or the abstract | 1 |
| (b) Provide in the abstract an informative and balanced summary of what was done and what was found | 1 |
| **Introduction** |  |
| Background/rationale |  |
| Explain the scientific background and rationale for the investigation being reported | 1, 2 |
| Objectives |  |
| State specific objectives, including any pre specified hypotheses | 2 |
| **Methods** |  |
| Study design |  |
| Present key elements of study design early in the paper | N |
| Setting |  |
| Describe the setting, locations, and relevant dates, including periods of recruitment, exposure, follow-up, and data collection | 2, 3 |
| Participants |  |
| (a) Cohort study—give the eligibility criteria, and the sources and methods of selection of participants. Describe methods of follow-up | 2, 3 |
| Cross-sectional study—give the eligibility criteria, and the sources and methods of selection of participants | NA |
| (b) Cohort study—for matched studies, give matching criteria and number of exposed and unexposed | N |
| Variables |  |
| Clearly define all outcomes, exposures, predictors, potential confounders, and effect modifiers. Give diagnostic criteria, if applicable | 3, 4 |
| Data sources/ measurement |  |
| For each variable of interest give sources of data and details of methods of assessment (measurement). Describe comparability of assessment methods if there is more than one group | 2, 3, 4, 5 |
| Bias |  |
| Describe any efforts to address potential sources of bias | N |
| Study size |  |
| Explain how the study size was arrived at | 2, 3 |
| Quantitative variables |  |
| Explain how quantitative variables were handled in the analyses. If applicable, describe which groupings were chosen, and why | 3, 4, 5 |
| Statistical methods |  |
| (a) Describe all statistical methods, including those used to control for confounding | N |
| (b) Describe any methods used to examine subgroups and interactions | 3, 4, 5 |
| (c) Explain how missing data were addressed | N |
| (d) Cohort study—if applicable, explain how loss to follow-up was addressed | N |
| Cross-sectional study—if applicable, describe analytical methods taking account of sampling strategy | NA |
| (e) Describe any sensitivity analyses | 4, 5 |
| **Results** |  |
| Participants |  |
| (a) Report the numbers of individuals at each stage of the study—eg, numbers potentially eligible, examined for eligibility, confirmed eligible, included in the study, completing follow-up, and analyzed | 2, 3 |
| (b) Give reasons for non-participation at each stage | N |
| (c) Consider use of a flow diagram | N |
| Descriptive data |  |
| (a) Give characteristics of study participants (eg, demographic, clinical, social) and information on exposures and potential confounders | 2, 3, 5 |
| (b) Indicate the number of participants with missing data for each variable of interest | N |
| (c) Cohort study—summarize follow-up time (eg, average and total amount) | N |
| Outcome data |  |
| Cohort study—report numbers of outcome events or summary measures over time | 5, 6, 7 |
| Cross-sectional study—report numbers of outcome events or summary measures | NA |
| Main results |  |
| (a) Give unadjusted estimates and, if applicable, confounder-adjusted estimates and their precision (eg, 95% confidence interval). Make clear which confounders were adjusted for and why they were included | 5, 6, 7 |
| (b) Report category boundaries when continuous variables were categorized | N |
| (c) If relevant, consider translating estimates of relative risk into absolute risk for a meaningful time period | NA |
| Other analyses |  |
| Report other analyses done—eg, analyses of subgroups and interactions, and sensitivity analyses | 6, 7 |
| **Discussion** |  |
| Key results |  |
| Summarize key results with reference to study objectives | 7, 8 |
| Limitations |  |
| Discuss limitations of the study, taking into account sources of potential bias or imprecision. Discuss both direction and magnitude of any potential bias | 9 |
| Interpretation |  |
| Give a cautious overall interpretation of results considering objectives, limitations, multiplicity of analyses, results from similar studies, and other relevant evidence | 7, 8, 9 |
| Generalisability |  |
| Discuss the generalisability (external validity) of the study results | 9 |
| **Other information** |  |
| Funding |  |
| Give the source of funding and the role of the funders for the present study and, if applicable, for the original study on which the present article is based | 9 |

N, No; NA, Not Available.

**Supplementary Table SM7.** STROBE Statement Rosi A, 2020

| **Item Recommendation Reported on manuscript** | **Page** |
| --- | --- |
| **Title and abstract** |  |
| (a) Indicate the study’s design with a commonly used term in the title or the abstract | 1 |
| (b) Provide in the abstract an informative and balanced summary of what was done and what was found | 1 |
| **Introduction** |  |
| Background/rationale |  |
| Explain the scientific background and rationale for the investigation being reported | 1, 2 |
| Objectives |  |
| State specific objectives, including any pre specified hypotheses | N |
| **Methods** |  |
| Study design |  |
| Present key elements of study design early in the paper | N |
| Setting |  |
| Describe the setting, locations, and relevant dates, including periods of recruitment, exposure, follow-up, and data collection | 2, 3 |
| Participants |  |
| (a) Cohort study—give the eligibility criteria, and the sources and methods of selection of participants. Describe methods of follow-up | 2, 3 |
| Cross-sectional study—give the eligibility criteria, and the sources and methods of selection of participants | NA |
| (b) Cohort study—for matched studies, give matching criteria and number of exposed and unexposed | N |
| Variables |  |
| Clearly define all outcomes, exposures, predictors, potential confounders, and effect modifiers. Give diagnostic criteria, if applicable | 2, 3, 4 |
| Data sources/ measurement |  |
| For each variable of interest give sources of data and details of methods of assessment (measurement). Describe comparability of assessment methods if there is more than one group | 2, 3, 4 |
| Bias |  |
| Describe any efforts to address potential sources of bias | 3, 4 |
| Study size |  |
| Explain how the study size was arrived at | 2, 3 |
| Quantitative variables |  |
| Explain how quantitative variables were handled in the analyses. If applicable, describe which groupings were chosen, and why | 3, 4 |
| Statistical methods |  |
| (a) Describe all statistical methods, including those used to control for confounding | 3, 4 |
| (b) Describe any methods used to examine subgroups and interactions | 3, 4 |
| (c) Explain how missing data were addressed | 2 |
| (d) Cohort study—if applicable, explain how loss to follow-up was addressed | 2 |
| Cross-sectional study—if applicable, describe analytical methods taking account of sampling strategy | NA |
| (e) Describe any sensitivity analyses | 3, 4 |
| **Results** |  |
| Participants |  |
| (a) Report the numbers of individuals at each stage of the study—eg, numbers potentially eligible, examined for eligibility, confirmed eligible, included in the study, completing follow-up, and analyzed | 2, 3 |
| (b) Give reasons for non-participation at each stage | 2 |
| (c) Consider use of a flow diagram | N |
| Descriptive data |  |
| (a) Give characteristics of study participants (eg, demographic, clinical, social) and information on exposures and potential confounders | 2 |
| (b) Indicate the number of participants with missing data for each variable of interest | N |
| (c) Cohort study—summarize follow-up time (eg, average and total amount) | 4 |
| Outcome data |  |
| Cohort study—report numbers of outcome events or summary measures over time | 4, 5 |
| Cross-sectional study—report numbers of outcome events or summary measures | NA |
| Main results |  |
| (a) Give unadjusted estimates and, if applicable, confounder-adjusted estimates and their precision (eg, 95% confidence interval). Make clear which confounders were adjusted for and why they were included | 4, 5, 6, 7, 8, 9 |
| (b) Report category boundaries when continuous variables were categorized | N |
| (c) If relevant, consider translating estimates of relative risk into absolute risk for a meaningful time period | NA |
| Other analyses |  |
| Report other analyses done—eg, analyses of subgroups and interactions, and sensitivity analyses | 6, 7 |
| **Discussion** |  |
| Key results |  |
| Summarize key results with reference to study objectives | 9 |
| Limitations |  |
| Discuss limitations of the study, taking into account sources of potential bias or imprecision. Discuss both direction and magnitude of any potential bias | 10, 11 |
| Interpretation |  |
| Give a cautious overall interpretation of results considering objectives, limitations, multiplicity of analyses, results from similar studies, and other relevant evidence | 9, 10, 11 |
| Generalisability |  |
| Discuss the generalisability (external validity) of the study results | 10, 11 |
| **Other information** |  |
| Funding |  |
| Give the source of funding and the role of the funders for the present study and, if applicable, for the original study on which the present article is based | NA |

N, No; NA, Not Available.

**Supplementary Table SM8.** STROBE Statement Telleria-Aramburu N, 2021

| **Item Recommendation Reported on manuscript** | **Page** |
| --- | --- |
| **Title and abstract** |  |
| (a) Indicate the study’s design with a commonly used term in the title or the abstract | 1 |
| (b) Provide in the abstract an informative and balanced summary of what was done and what was found | 1 |
| **Introduction** |  |
| Background/rationale |  |
| Explain the scientific background and rationale for the investigation being reported | 1, 2 |
| Objectives |  |
| State specific objectives, including any pre specified hypotheses | 2, 4 |
| **Methods** |  |
| Study design |  |
| Present key elements of study design early in the paper | 2 |
| Setting |  |
| Describe the setting, locations, and relevant dates, including periods of recruitment, exposure, follow-up, and data collection | 2, 3 |
| Participants |  |
| (a) Cohort study—give the eligibility criteria, and the sources and methods of selection of participants. Describe methods of follow-up | NA |
| Cross-sectional study—give the eligibility criteria, and the sources and methods of selection of participants | 2, 3 |
| (b) Cohort study—for matched studies, give matching criteria and number of exposed and unexposed | NA |
| Variables |  |
| Clearly define all outcomes, exposures, predictors, potential confounders, and effect modifiers. Give diagnostic criteria, if applicable | 2, 3, 4, 6 |
| Data sources/ measurement |  |
| For each variable of interest give sources of data and details of methods of assessment (measurement). Describe comparability of assessment methods if there is more than one group | 2, 3, 4, 6 |
| Bias |  |
| Describe any efforts to address potential sources of bias | 4, 6 |
| Study size |  |
| Explain how the study size was arrived at | 2 |
| Quantitative variables |  |
| Explain how quantitative variables were handled in the analyses. If applicable, describe which groupings were chosen, and why | 2, 3, 4, 6 |
| Statistical methods |  |
| (a) Describe all statistical methods, including those used to control for confounding | 2, 3, 4, 6 |
| (b) Describe any methods used to examine subgroups and interactions | 2, 3, 4, 6 |
| (c) Explain how missing data were addressed | 3 |
| (d) Cohort study—if applicable, explain how loss to follow-up was addressed | NA |
| Cross-sectional study—if applicable, describe analytical methods taking account of sampling strategy | 2 |
| (e) Describe any sensitivity analyses | 4, 6 |
| **Results** |  |
| Participants |  |
| (a) Report the numbers of individuals at each stage of the study—eg, numbers potentially eligible, examined for eligibility, confirmed eligible, included in the study, completing follow-up, and analyzed | 2, 3 |
| (b) Give reasons for non-participation at each stage | 2 |
| (c) Consider use of a flow diagram | 2 |
| Descriptive data |  |
| (a) Give characteristics of study participants (eg, demographic, clinical, social) and information on exposures and potential confounders | 2 |
| (b) Indicate the number of participants with missing data for each variable of interest | N |
| (c) Cohort study—summarize follow-up time (eg, average and total amount) | NA |
| Outcome data |  |
| Cohort study—report numbers of outcome events or summary measures over time | NA |
| Cross-sectional study—report numbers of outcome events or summary measures | 4, 5, 6, 7 |
| Main results |  |
| (a) Give unadjusted estimates and, if applicable, confounder-adjusted estimates and their precision (eg, 95% confidence interval). Make clear which confounders were adjusted for and why they were included | 4, 5, 6, 7 |
| (b) Report category boundaries when continuous variables were categorized | N |
| (c) If relevant, consider translating estimates of relative risk into absolute risk for a meaningful time period | NA |
| Other analyses |  |
| Report other analyses done—eg, analyses of subgroups and interactions, and sensitivity analyses | 4, 5, 6, 7 |
| **Discussion** |  |
| Key results |  |
| Summarize key results with reference to study objectives | 7 |
| Limitations |  |
| Discuss limitations of the study, taking into account sources of potential bias or imprecision. Discuss both direction and magnitude of any potential bias | 9 |
| Interpretation |  |
| Give a cautious overall interpretation of results considering objectives, limitations, multiplicity of analyses, results from similar studies, and other relevant evidence | 7, 8, 9 |
| Generalisability |  |
| Discuss the generalisability (external validity) of the study results | 8, 9 |
| **Other information** |  |
| Funding |  |
| Give the source of funding and the role of the funders for the present study and, if applicable, for the original study on which the present article is based | 9 |

N, No; NA, Not Available.

**Supplementary Table SM9.** STROBE Statement Naja F, 2022

| **Item Recommendation Reported on manuscript** | **Page** |
| --- | --- |
| **Title and abstract** |  |
| (a) Indicate the study’s design with a commonly used term in the title or the abstract | N |
| (b) Provide in the abstract an informative and balanced summary of what was done and what was found | 1 |
| **Introduction** |  |
| Background/rationale |  |
| Explain the scientific background and rationale for the investigation being reported | 1, 2 |
| Objectives |  |
| State specific objectives, including any pre specified hypotheses | N |
| **Methods** |  |
| Study design |  |
| Present key elements of study design early in the paper | N |
| Setting |  |
| Describe the setting, locations, and relevant dates, including periods of recruitment, exposure, follow-up, and data collection | 2, 3 |
| Participants |  |
| (a) Cohort study—give the eligibility criteria, and the sources and methods of selection of participants. Describe methods of follow-up | NA |
| Cross-sectional study—give the eligibility criteria, and the sources and methods of selection of participants | 2, 3 |
| (b) Cohort study—for matched studies, give matching criteria and number of exposed and unexposed | NA |
| Variables |  |
| Clearly define all outcomes, exposures, predictors, potential confounders, and effect modifiers. Give diagnostic criteria, if applicable | 2, 3, 4 |
| Data sources/ measurement |  |
| For each variable of interest give sources of data and details of methods of assessment (measurement). Describe comparability of assessment methods if there is more than one group | 2, 3, 4 |
| Bias |  |
| Describe any efforts to address potential sources of bias | 4, 11 |
| Study size |  |
| Explain how the study size was arrived at | 2, 3 |
| Quantitative variables |  |
| Explain how quantitative variables were handled in the analyses. If applicable, describe which groupings were chosen, and why | 3, 4 |
| Statistical methods |  |
| (a) Describe all statistical methods, including those used to control for confounding | 2, 3, 4 |
| (b) Describe any methods used to examine subgroups and interactions | 3, 4 |
| (c) Explain how missing data were addressed | 11 |
| (d) Cohort study—if applicable, explain how loss to follow-up was addressed | NA |
| Cross-sectional study—if applicable, describe analytical methods taking account of sampling strategy | 2, 3 |
| (e) Describe any sensitivity analyses | 4 |
| **Results** |  |
| Participants |  |
| (a) Report the numbers of individuals at each stage of the study—eg, numbers potentially eligible, examined for eligibility, confirmed eligible, included in the study, completing follow-up, and analyzed | 2, 3 |
| (b) Give reasons for non-participation at each stage | 2 |
| (c) Consider use of a flow diagram | N |
| Descriptive data |  |
| (a) Give characteristics of study participants (eg, demographic, clinical, social) and information on exposures and potential confounders | 2, 5 |
| (b) Indicate the number of participants with missing data for each variable of interest | N |
| (c) Cohort study—summarize follow-up time (eg, average and total amount) | NA |
| Outcome data |  |
| Cohort study—report numbers of outcome events or summary measures over time | NA |
| Cross-sectional study—report numbers of outcome events or summary measures | 4, 5, 6, 7 |
| Main results |  |
| (a) Give unadjusted estimates and, if applicable, confounder-adjusted estimates and their precision (eg, 95% confidence interval). Make clear which confounders were adjusted for and why they were included | 4, 5, 6, 7 |
| (b) Report category boundaries when continuous variables were categorized | N |
| (c) If relevant, consider translating estimates of relative risk into absolute risk for a meaningful time period | NA |
| Other analyses |  |
| Report other analyses done—eg, analyses of subgroups and interactions, and sensitivity analyses | 6, 7, 8, 9, 10 |
| **Discussion** |  |
| Key results |  |
| Summarize key results with reference to study objectives | 7, 10 |
| Limitations |  |
| Discuss limitations of the study, taking into account sources of potential bias or imprecision. Discuss both direction and magnitude of any potential bias | 11 |
| Interpretation |  |
| Give a cautious overall interpretation of results considering objectives, limitations, multiplicity of analyses, results from similar studies, and other relevant evidence | 7, 10, 11 |
| Generalisability |  |
| Discuss the generalisability (external validity) of the study results | 11 |
| **Other information** |  |
| Funding |  |
| Give the source of funding and the role of the funders for the present study and, if applicable, for the original study on which the present article is based | 12 |

N, No; NA, Not Available.

**Supplementary Table SM10.** STROBE Statement Tepper S, 2022

| **Item Recommendation Reported on manuscript** | **Page** |
| --- | --- |
| **Title and abstract** |  |
| (a) Indicate the study’s design with a commonly used term in the title or the abstract | N |
| (b) Provide in the abstract an informative and balanced summary of what was done and what was found | 1, 2 |
| **Introduction** |  |
| Background/rationale |  |
| Explain the scientific background and rationale for the investigation being reported | 2 |
| Objectives |  |
| State specific objectives, including any pre specified hypotheses | N |
| **Methods** |  |
| Study design |  |
| Present key elements of study design early in the paper | N |
| Setting |  |
| Describe the setting, locations, and relevant dates, including periods of recruitment, exposure, follow-up, and data collection | 2, 3, 4 |
| Participants |  |
| (a) Cohort study—give the eligibility criteria, and the sources and methods of selection of participants. Describe methods of follow-up | NA |
| Cross-sectional study—give the eligibility criteria, and the sources and methods of selection of participants | 2 |
| (b) Cohort study—for matched studies, give matching criteria and number of exposed and unexposed | NA |
| Variables |  |
| Clearly define all outcomes, exposures, predictors, potential confounders, and effect modifiers. Give diagnostic criteria, if applicable | 2, 3, 4 |
| Data sources/ measurement |  |
| For each variable of interest give sources of data and details of methods of assessment (measurement). Describe comparability of assessment methods if there is more than one group | 2, 3, 4, 5 |
| Bias |  |
| Describe any efforts to address potential sources of bias | 3, 4 |
| Study size |  |
| Explain how the study size was arrived at | 2 |
| Quantitative variables |  |
| Explain how quantitative variables were handled in the analyses. If applicable, describe which groupings were chosen, and why | 2, 3, 4, 5 |
| Statistical methods |  |
| (a) Describe all statistical methods, including those used to control for confounding | 2, 3, 4, 5 |
| (b) Describe any methods used to examine subgroups and interactions | 2, 3, 4, 5 |
| (c) Explain how missing data were addressed | 3 |
| (d) Cohort study—if applicable, explain how loss to follow-up was addressed | NA |
| Cross-sectional study—if applicable, describe analytical methods taking account of sampling strategy | N |
| (e) Describe any sensitivity analyses | 4, 5 |
| **Results** |  |
| Participants |  |
| (a) Report the numbers of individuals at each stage of the study—eg, numbers potentially eligible, examined for eligibility, confirmed eligible, included in the study, completing follow-up, and analyzed | 2, 5 |
| (b) Give reasons for non-participation at each stage | 3 |
| (c) Consider use of a flow diagram | N |
| Descriptive data |  |
| (a) Give characteristics of study participants (eg, demographic, clinical, social) and information on exposures and potential confounders | 4 |
| (b) Indicate the number of participants with missing data for each variable of interest | N |
| (c) Cohort study—summarize follow-up time (eg, average and total amount) | NA |
| Outcome data |  |
| Cohort study—report numbers of outcome events or summary measures over time | NA |
| Cross-sectional study—report numbers of outcome events or summary measures | 4, 5, 6, 7, 8 |
| Main results |  |
| (a) Give unadjusted estimates and, if applicable, confounder-adjusted estimates and their precision (eg, 95% confidence interval). Make clear which confounders were adjusted for and why they were included | 4, 5, 6, 7, 8 |
| (b) Report category boundaries when continuous variables were categorized | N |
| (c) If relevant, consider translating estimates of relative risk into absolute risk for a meaningful time period | NA |
| Other analyses |  |
| Report other analyses done—eg, analyses of subgroups and interactions, and sensitivity analyses | 5, 6, 7, 8 |
| **Discussion** |  |
| Key results |  |
| Summarize key results with reference to study objectives | 7 |
| Limitations |  |
| Discuss limitations of the study, taking into account sources of potential bias or imprecision. Discuss both direction and magnitude of any potential bias | 9 |
| Interpretation |  |
| Give a cautious overall interpretation of results considering objectives, limitations, multiplicity of analyses, results from similar studies, and other relevant evidence | 7, 8, 9 |
| Generalisability |  |
| Discuss the generalisability (external validity) of the study results | 9 |
| **Other information** |  |
| Funding |  |
| Give the source of funding and the role of the funders for the present study and, if applicable, for the original study on which the present article is based | 10 |

N, No; NA, Not Available.

**Supplementary Table SM11.** Indexes evaluating the adherence to the Mediterranean diet

| **Reference** | **Index name** | **Composition of MD (food or nutrient components)** | **Scoring or cut-off** | **Dietary assessment** |
| --- | --- | --- | --- | --- |
| Trichopoulou et al., 2003 | MDS a priori | 9: (+) V, legumes, f and nuts, cereals, fish. (-) Meat, dairy products, Moderate OH (10–50 g/day for men; 5–25 g/day for women); ratio MUFA-SFA. | 0–9 | Semi-quantitative validated FFQ (136 FI); FFQ (110 FI); FFQ (116 FI) |
| Struijk et al., 2014 | mMDS a priori | Same as MDS except for the ratio MUFA to PUFA over SFA. | 0–9 | 4-day food diaries |
| Naja et al., 2015 | Lebanese-MDScore a posteriori | 9: F, v, legumes, olive oil, burghol (crushed whole wheat), milk and dairy products, starchy vegetables (including potato, corn and peas), dried fruits and eggs. | 9–27 | FFQ (61 FI) |
| Fung et al., 2009 | aMed Greece | 9: MUFA-SFA ratio, v (excluding potatoes), f, nuts, legumes, whole grains, fish, alcohol, red meat, processed meat. | 0–9 | FFQ (61 FI) |
| Buckland et al., 2009 | rMed Spain | 9: (+) Olive oil, v (excluding potatoes), f, nuts, seeds (excluding fruit juices), cereals, legumes, fresh fish, alcohol (moderately). (-) Meat, meat products, dairy products. | 0–18 | FFQ (61 FI) |
| Gerber, 2006 | Med-DQI France | 7: (+) Olive oil, fish, cereals, v, f. (-) Meats, SFA, cholesterol. | 0–14 | FFQ (61 FI) |
| Aparicio-Ugarriza et al., 2019 (HELENA study) | Adaptation of MDS Trichopoulou et al., 2003 a priori | 7: (+) V, legumes, f and nuts, cereals, fish, MUFA-SFA ratio, and wine. 2: (-) Meat and poultry, and dairy products. | Adherence to the MD: low (score 0–3 points), medium (4–5 points), and high (6–9 points) | Winter and spring: 3-consecutive days dietary record each season |
| Panagiotakos et al., 2006 | MDS a priori | 11: Olive oil, v, potatoes, legumes, f, non-refined cereals, fish, alcohol, red meat, poultry, and full fat dairy products. | 0 - 55 | FFQ (67 FI) |
| Naja et al., 2022 | c-MED Index a priori | 8: (+) Whole grains, fish, legumes, f, v, olive oil-to-SFA ratio. (-) Refined grains, sugar sweetened beverages. | 0 - 8 | Collection of dietary intake with a single 24h recall |

aMed, Alternate Mediterranean diet score; c-MED, Composite Mediterranean; F, Fruit; FFQ, Food frequency questionnaire; FI, Food Items; g, grams; LMD, Lebanese-Mediterranean diet; MDS, Mediterranean Diet Score; Med-DQI, Mediterranean Diet Quality Index; mMDS, Modified Mediterranean diet score; MUFA, Monounsaturated fats; OH, Alcohol; PUFA, Polyunsaturated fats; rMed, Spain Mediterranean Index; SFA, Saturated fatty acids; V, Vegetables; (+), Positive; (-), Negative.

**Supplementary Table SM12.** Indexes evaluating the health aspect of the diets of the articles included

| **First author, year** | **Method used to measure the nutrients quality** | **Result of nutrients quality** | **MD Definition** | **Diets included in the study (apart of MD)** | **Main conclusions** |
| --- | --- | --- | --- | --- | --- |
| Van Dooren C, 2014 | Health Score (W) | 122 | Dutch Dietary Guidelines: Health Council, 2006. | Average Dutch; DDG Semi-vegetarian; Traditional vegetarian; Vegan | Mediterranean diet had the higher nutritional quality |
| Van Dooren C, 2016 | Health Score (M) | 122 | Dutch Dietary Guidelines: Health Council, 2006. | NND, traditional LLD, Dietary guideline and Optimised LLD, present Dutch diets | An optimized LLD has the same healthy nutritional characteristics as MD, but less than NND |
| Castañé S, 2017 | Nutrient Rich Foods Index | 90.6 | Bach-Faig et al., 2011 | VD (103) | VD had a higher nutritional quality than MD |
| Chapa J, 2020 | Nutrient Rich Foods Index, Nutritional Quality Index | 13.51, 107.27 | The sample 2-week menu: USDA ChooseMyPlate guide and the work of Migala, 2019 | Healthy U.S., VEG, and “typical” diets | The highest NRF values were observed in diets that included dairy within the healthy U.S. and vegetarian diets. Meanwhile, the highest NQI was associated with HUS and “typical” diets |
| Cambeses-Franco C, 2022 | Nutrient Rich Diet Index, Health gain score | 477, 178 | González-García et al., 2020 | NAOS, NND, DDG, DGA, IDG | MD clearly had the highest NRD 9.3 health gain score |
| Paris JMG, 2022 | Human health indicators | VD<MD<DD<RD* | Bach-Faig et al., 2011; Fidanza F, Alberti A, 2005 | The national dietary guidelines, VD | VD causes the lowest exposure to dietary risk factors and hence the smallest impact among all diets. However, there is still the risk of developing hypertensive heart disease and stomach cancer |

*Results by diets according to lowest disability-adjusted life years due to non-communicable diseases. DD, German Nutrition Society diet; DDG, Dutch Dietary Guidelines; DGA, American Dietary Guidelines; HUS, Healthy U.S.; IDG, Italian Dietary Guidelines; LLD, Low Lands Diet; M, Man; MD, Mediterranean Diet; NAOS, Spanish dietary guidelines; NND, New Nordic Diet; NQI, Nutritional Quality Idex; NRD, Nutrient Rich Diet; NRF, Nutrient Rich Foods; RD, Reference Diet; VD, Vegan Diet; VEG, Vegetarian Diet; W, Woman.

**Supplementary Table SM13.** System boundary of the articles included

| **Article** | **System boundary** | | | | |
| --- | --- | --- | --- | --- | --- |
|  | From cradle-to-farm gate | From cradle-to-manufacture gate | From cradle-to-retail | From cradle-to-consumer | From cradle-to-waste |
| Tukker A, 2011 |  |  | X |  |  |
| Sáez-Almendros S, 2013 |  |  | X |  |  |
| Wilson N, 2013 | X |  |  |  |  |
| Capone R, 2013 |  |  |  |  |  |
| Germani A, 2014 |  |  | X |  |  |
| Van Dooren C, 2014 | X |  |  |  |  |
| Vanham D, 2014 | X |  |  |  |  |
| Tilman D, 2014 | X |  |  |  |  |
| Pairotti MB, 2014 |  |  |  | X |  |
| Vidal R, 2015 |  |  | X |  |  |
| Van Dooren C, 2016 | X |  |  |  |  |
| Benvenuti L, 2016 | X |  |  |  |  |
| Blas A, 2016 | X |  |  |  |  |
| Vanham D, 2016 | X |  |  |  |  |
| Castañé S, 2017 |  |  |  | X |  |
| Ulaszewska MM, 2017 |  |  |  | X |  |
| Fresán U, 2018 |  | X |  |  |  |
| Murakami K, 2018 |  |  |  |  | X |
| Blackstone NT, 2018 |  |  |  | X |  |
| Naja F, 2018 |  | X |  |  |  |
| Blas A, 2019 | X |  |  |  |  |
| Batlle-Bayer L, 2019 |  |  |  | X |  |
| Naja F, 2019 |  |  |  |  |  |
| Chapa J, 2020 |  | X |  |  |  |
| Grosso G, 2020 |  | X |  |  |  |
| González-García S, 2020 |  |  |  | X |  |
| Rosi A, 2020 |  |  |  | X |  |
| Belgacem W, 2021 | X |  |  |  |  |
| Telleria-Aramburu N, 2021 |  |  |  |  | X |
| Vanham D, 2021 | X |  |  |  |  |
| Cambeses-Franco C, 2022 |  | X |  |  |  |
| Paris JMG, 2022 |  |  |  | X |  |
| Naja F, 2022 |  |  | X |  |  |
| Castaldi S, 2022 |  |  | X |  |  |
| Tepper S, 2022 |  |  |  | X |  |

**Supplementary Table SM14.** Nutritional considerations in the functional unit of the articles included

| **Article** | **Nutritional considerations in functional unit** | | | | | |
| --- | --- | --- | --- | --- | --- | --- |
|  | Energy content |  | Protein content | Fat content | Carbohydrates content | Grams |
| Tukker A, 2011 | X |  | X | X |  |  |
| Sáez-Almendros S, 2013 |  |  |  |  |  | X |
| Wilson N, 2013 |  |  |  |  |  | X |
| Capone R, 2013 |  |  |  |  |  | X |
| Germani A, 2014 | X |  | X | X | X |  |
| Van Dooren C, 2014 |  |  |  |  |  | X |
| Vanham D, 2014 |  |  |  |  |  | X |
| Tilman D, 2014 | X |  | X |  |  | X |
| Pairotti MB, 2014 |  |  |  |  |  | X |
| Vidal R, 2015 |  |  |  |  |  | X |
| Van Dooren C, 2016 |  |  |  |  |  | X |
| Benvenuti L, 2016 |  |  |  |  |  | X |
| Blas A, 2016 |  |  |  |  |  | X |
| Vanham D, 2016 |  |  |  |  |  | X |
| Castañé S, 2017 | X |  |  |  |  | X |
| Ulaszewska MM, 2017 |  |  |  |  |  | X |
| Fresán U, 2018 |  |  |  |  |  |  |
| Murakami K, 2018 |  |  |  |  |  | X |
| Blackstone NT, 2018 |  |  |  |  |  | X |
| Naja F, 2018 |  |  |  |  |  | X |
| Blas A, 2019 |  |  |  |  |  | X |
| Batlle-Bayer L, 2019 | X |  |  |  |  | X |
| Naja F, 2019 |  |  |  |  |  |  |
| Chapa J, 2020 |  |  |  |  |  | X |
| Grosso G, 2020 |  |  |  |  |  | X |
| González-García S, 2020 |  |  |  |  |  | X |
| Rosi A, 2020 |  |  |  |  |  | X |
| Belgacem W, 2021 |  |  |  |  |  | X |
| Telleria-Aramburu N, 2021 |  |  |  |  |  | X |
| Vanham D, 2021 |  |  |  |  |  | X |
| Cambeses-Franco C, 2022 |  |  |  |  |  | X |
| Paris JMG, 2022 | X |  |  |  |  | X |
| Naja F, 2022 |  |  |  |  |  | X |
| Castaldi S, 2022 |  |  |  |  |  | X |
| Tepper S, 2022 |  |  |  |  |  | X |

**Supplementary Table SM15.** Environmental footprint of MD’s foods or food groups

| **First author, year, and location** | **MD Definition** | **Unit of measure** | **GHGE** | **Water footprint** | **Land use** |
| --- | --- | --- | --- | --- | --- |
| Sáez-Almendros S, 2013, Spain | Bach-Faig et al., 2011 | GHGE- kg CO2-eq/capita-year, WF- l/capita-year, LU- m2/capita-year. | Dairy 439.6, Meat 211.1, Fish 75.1, Vegetal oils and fats 42.6, Nuts 8.7, Eggs 5.9, Vegetables 4.1, Cereals 3.8, Fruit 2, Tubers 1.5, Sugar and Sweets 0. | Vegetal oils and fats 42740.88, Dairy 41797.82, Nuts 12439.7, Meat 7800, Eggs 2426.84, Vegetables 1822.24, Tubers 300.3, Fish 287.28, Fruit 1.01, Cereals 0.09, Sweets and sugar 0. | Dairy 715, Meat 309, Cereals 217, Fruit 207, Nuts 200, Vegetal oils and fats 133, Vegetables 84, Eggs 4, Tubers 3, Sweets and sugar 0, Fish not applicable. |
| Wilson N, 2013, New Zealand | Trichopoulou et al., 2009 | kg CO2e/ 100g | *Fruit and vegetables 2.35, Cereals and grains 0.8, Dairy products 0.36, Other foods 0.24, Fish and meat 0.14, Pulses, seeds and nuts 0.02. |  |  |
| Capone R, 2013, Italy | Italian Institute of Food Science of the La Sapienza University-Rome, 2006 | m3/capita/year |  | Meat 732.313, Vegetable Oils 268.72, Milk (excluding butter) 225.872, Cereals (excluding beer) 203.41, Stimulants 126.333, Fruits (excluding wine) 66.905, Sugar & Sweeteners 57.83, Pulses 57.83, Alcoholic Beverages 36.781, Animal Fats 34.71, Vegetables 34.555, Offals 18.697, Eggs 14.878, Oilcrops 10.301, Starchy Roots 7.78, Spices 1.219, Fish, Seafood 0, Sugarcrops 0. |  |
| Vanham D, 2014, Italy | Bach-Faig et al., 2011 | l/cap/d |  | Meat 775.6, Cereals, rice, potatoes 723.8, Milk and milkproducts 453.3, Crop oils 448.3, Non edible agr. products 378.6, Stimulants 333.8, Fruit 189.6, Pulses, nuts and oilcrops 164.5, Sugar 122.3, Vegetables 116.6, Alcoholic beverages 85.4, Eggs 43.5, Spices 4. |  |
| Vidal R, 2015, Spain | Diet published by Benidorm Clinical Hospital | kg CO2 eq/day | 120 g meat or 150 g fish or 2 eggs 1.620, 130 g rice/pasta or 450 g potatoes 1.323, 120 g meat or 150 g fish or 2 eggs 0.728, 200 cc milk with coffee 0.324, 200 cc milk 0.210, 130 g fruit 0.160, 200 g vegetables 0.148, 200 g vegetables/salad 0.142, 180 g bread 0.141, 130 g fruit 0.136, 40 g biscuits 0.106, 20 g marmalade 0.016, 15 g butter 0.010, 20g sugar 0.018. |  |  |
| Blas A, 2016, Spain | Bach-Faig et al., 2011 | l/person-day |  | Rest 2847, Olive oil 1055, Milk 475, Eggs 316, Beef meat 316, Cheese 264. |  |
| Castañé S, 2017, Mediterranean Area | Bach-Faig et al., 2011, (41), with weekly diet composed of 7 daily menus | kg CO2eq/kg product | Dairy 30.5, Fish 21.5, Meat 17.2, Grains 7.7, Nuts/Seeds 3.2, Fats/Oils 2.5, Fruits 2.4, Vegetables 2.32, Egg 2, Legumes 1.9, Soy products 1.8, Sweets 1.4. |  |  |
| Ulaszewska MM, 2017, Italy | INRAN guidelines, 2003, (59). Consumption of tap and bottled water: EFSA Comprehensive European Food Consumption Database | kg CO2 eq/week | Milk and dairy products 5.54, Fruits and vegetables 5.32, Cereals and potatoes 4.2, Meat and cold cuts 3.14, Poultry, fish, legumes and eggs 2.54, Tap water 1.64, Herbs/oils/condiments 1.17. |  |  |
| Blackstone NT, 2018, USA | USA Dietary Guidelines Advisory Committee, 2015 | GHGE- kg CO2 eq, Water Depletion- m3 water eq, LU- kg C deficit | Protein 16.348, Dairy 3.585, Vegetable 1.313, Grain 1.021, Fruit 0.952, Extra 0.762, Oils 0.693. | Fruit 0.223, Vegetable 0.197, Protein 0.164, Grain 0.074, Dairy 0.073, Extra 0.018, Oils 0.001. | Protein 261.003, Grain 42.189, Dairy 38.006, Vegetable 25.518, Oils 12.307, Extra 10.300, Fruit 7.825. |
| Naja F, 2018, Lebanon | Lebanese-MD- Naja et al., 2015 | GHGE- % contribution (mean ± SD) kg CO2eq / day, WF- % contribution (mean ± SD) l/day | Vegetables 50.75 ± 21.08, Whole dairy products 22.02 ± 20.32, Fruits 10.23 ± 8.73, Legumes 6.90 ± 6.39, Olives 6.55 ± 10.04, Burghol (whole wheat parboiled and crushed) 2.16 ± 3.8, Traditional sweets 1.07 ± 1.55, Dried fruits 0.33 ± 1.19. | Whole dairy products 43.01 ± 19.05, Fruits 20.69 ± 14.09, Legumes 13.11 ± 9.59, Vegetables 10.29 ± 7.68, Olives 8.39 ± 11.33, Burghol (whole wheat parboiled and crushed) 2.13 ± 4.42, Traditional sweets 2.09 ± 2.89, Dried fruits 0.29 ± 1.06. |  |
| Blas A, 2019, Spain | Bach-Faig et al., 2011 | m3/ person and year |  | Olive Oil and healthy drinks 369.66, Dairy products 134.18, Fruits and vegetables 126.84, Olives, nuts, seeds and condiments 93.97, Eggs and legumes 65.56, Red or processed meat 52.18, Cereals, White Meat and Vegetable fats 34.83, Sugar, sweets, sauces and beverages 10.01, Fish and Seafood 0. |  |
| Batlle-Bayer L, 2019, Spain | Bach-Faig et al., 2011 | tCO2-eq/capita-year | *Dairy products 0.34, Vegetables 0.29, Meat 0.17, Fish 0.16. |  |  |
| Telleria-Aramburu N, 2021, Spain | Panagiotakos et al., 2006 | kg eCO2/1000 kcal per day | Red meat and deli meat 28.23, Fruit and vegetables 19.18, Milk and dairy products 12.14, Eggs and white meats 11.52, Fish and shellfish 9.57, Sweets and salted snacks 5.28, Starchy foods 4.82, Cheese 3.33, Non-alcoholic drinks 2.5, Oil and fat 2.22, Alcoholic drinks 1.55. |  |  |
| Cambeses-Franco C, 2022, Spain | González-García et al., 2020 | GHGE- kg CO2eq·person−1·day−1, WF- L·person−1  ·day−1. | Dairy products 27.73, Meat 19.55, Starch.based products 17.27, Fish and seafood 8.64, Oils and fats 7.73, Fruits 7.27. | Oils and fats 776, Dairy products 591, Meat 311, Fruits 211. |  |
| Castaldi S, 2022, Italy | Willet et al., 2019 | kg CO2eq kg−1 | Red meat 18.09, Butter 8.48, Fish 4.52, Cereals 1.19, Cheese 4.38, Poultry 3.88, Vegetable oil 3.27, Eggs 3.20, Dairy 2.05, Nuts&seeds nuts 1.10, Sugar equivalents in jam or honey 0.82, Legumes 0.49, Fruit 0.45, Vegetables 0.41, Potatoes 0.24. |  |  |

GHGE, Greenhouse gas emissions; LU, Land use; WF, Water footprint. *Data were calculated with information provided in the article. Articles not included in the table were for different reasons: Data were not available for references 58, 63, 78, and 118. For articles with references 30, 35, 41, 68, 75, 103, 115, data were given with figures in different colors but without detailed numbers, and it was not possible to calculate it.  Tilman D. (73) had data for general foods or food categories but did not specify for MD and it was not possible to calculate it. The same occurs with Fresan's article (89), that gives the percentage of contribution of food groups and the final results are from linear regression models. Benvenuti (81) specified data for recipes, not by food or food categories. Naja F., 2019 (99), reported general data for 61 food items, and included four different types of MD in the article. Rosi A. (106) has the data for two different seasons. Vanham D., 2016 (86) and 2021 (113), shows data available in proportion (%) for 13 cities and nine countries, respectively.

**Supplementary Table SM16.** Modeling assessment of articles included

| **Reference** | **1** | **2** | **3** | **4** | **5** | **6** | **7** | **8** | **9** | **10** | **11** | **12** | **Total** |
| --- | --- | --- | --- | --- | --- | --- | --- | --- | --- | --- | --- | --- | --- |
| Tukker A, 2011 | Y | Y | Y | Y | N | Y | Y | Y | Y | N | Y | N | 9 |
| Sáez-Almendros S | Y | N | Y | Y | Y | Y | Y | Y | Y | N | Y | N | 9 |
| Wilson N, 2013 | Y | Y | Y | Y | Y | Y | Y | Y | Y | Y | Y | N | 11 |
| Capone R, 2013 | Y | N | Y | Y | N | Y | Y | Y | Y | N | Y | N | 8 |
| Germani A, 2014 | Y | N | Y | Y | N | Y | Y | Y | Y | Y | Y | N | 9 |
| Van Dooren C, 2014 | Y | N | Y | Y | Y | Y | Y | Y | Y | N | Y | N | 9 |
| Vanham D, 2014 | Y | Y | Y | Y | Y | Y | Y | Y | Y | N | Y | N | 10 |
| Tilman D, 2014 | Y | N | Y | Y | Y | Y | Y | Y | Y | N | Y | N | 9 |
| Pairotti MB, 2014 | Y | Y | Y | Y | Y | Y | Y | Y | Y | Y | Y | N | 11 |
| Vidal R, 2015 | Y | N | Y | Y | N | N | N | Y | Y | N | Y | N | 6 |
| Van Dooren C, 2016 | Y | N | Y | Y | Y | Y | Y | Y | Y | N | Y | N | 9 |
| Benvenuti L, 2016 | Y | Y | Y | Y | Y | Y | Y | Y | Y | N | Y | Y | 11 |
| Blas A, 2016 | Y | Y | Y | Y | N | Y | Y | Y | Y | Y | Y | N | 10 |
| Vanham D, 2016 | Y | N | Y | Y | N | Y | Y | Y | Y | N | Y | N | 8 |
| Castañé S, 2017 | Y | Y | Y | Y | N | Y | Y | Y | Y | Y | Y | Y | 11 |
| Ulaszewska MM, 2017 | Y | Y | Y | Y | Y | Y | Y | Y | Y | Y | Y | N | 11 |
| Blackstone NT, 2018 | Y | N | Y | Y | N | Y | Y | Y | Y | N | Y | N | 8 |
| Blas A, 2019 | Y | N | Y | Y | Y | Y | Y | Y | Y | Y | Y | N | 10 |
| Batlle-Bayer, 2019 | Y | N | Y | Y | Y | Y | Y | Y | Y | Y | Y | N | 10 |
| Chapa J, 2020 | Y | N | Y | Y | Y | Y | Y | Y | Y | N | Y | N | 9 |
| González-García S, 2020 | Y | N | Y | Y | Y | Y | Y | Y | Y | N | Y | N | 9 |
| Belgacem W, 2021 | Y | N | Y | Y | Y | Y | Y | Y | Y | N | Y | X | 10 |
| Vanham D, 2021 | Y | N | Y | Y | Y | Y | Y | Y | Y | Y | Y | N | 10 |
| Cambeses-Franco C, 2022 | Y | Y | Y | Y | N | Y | Y | Y | Y | Y | Y | N | 10 |
| Paris JMG, 2022 | Y | N | Y | Y | Y | Y | Y | Y | Y | Y | Y | N | 10 |
| Castaldi S, 2022 | Y | N | Y | Y | Y | Y | Y | Y | Y | N | Y | N | 9 |

N, No; Y, Yes. 1. A statement of the problem; 2. A discussion of the need for modeling vs. alternative methodologies; 3. A description of the relevant factors and outcomes; 4. A description of the model including reasons for this type of model and a specification of the scope including boundary conditions for analysis; 5. A listing of data sources (including subjective estimates), with a description of the strengths and weaknesses of each source; 6. A list of assumptions pertaining to the structure of the model and the data; 7. A list of parameter values that will be used for a base case analysis; 8. The results derived from applying the model for the base case; 9. A discussion of how the modeling assumptions might affect the results, indicating both the direction of the bias and the approximate magnitude of the effect; 10. A description of the validation undertaken, including the following where appropriate: concurrence of experts, internal consistency, external consistency, or predictive validity; 11. A description of the settings to which the results of the analysis can be applied and a list of factors that could limit the applicability of the results; 12. A description of research in progress that could yield new data that could alter the results of the analysis.
